# Anthraquinone Residues in Dried Walnut (*Juglans regia*) Leaves for Herbal Infusions: Proof of Endogenous Origin via a Sampling-Driven and GC-MS/MS-Based Strategy

**DOI:** 10.1021/acs.jafc.4c08102

**Published:** 2024-11-20

**Authors:** Lucas Ferrando Plo, Athanasios Nitsopoulos, Albrecht Friedle, Andreas Schmidberger, Jörg Heilmann

**Affiliations:** † Labor Friedle GmbH, Von-Heyden-Straße 11, 93105 Tegernheim, Germany; ‡ Institute of Pharmacy, University of Regensburg, Universitätsstraße 31, 93053 Regensburg, Germany

**Keywords:** anthraquinone, walnut, Juglans regia, polyketide, shikimic acid, sampling, QuEChERS, leaves, moss, GC-MS/MS

## Abstract

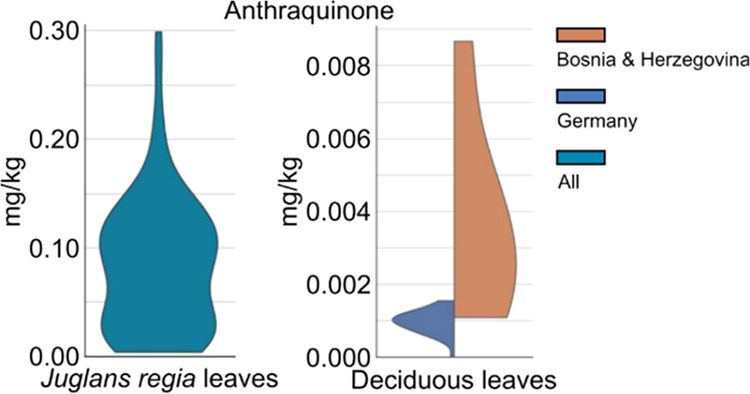

Anthraquinone residues in tea have been linked to atmospheric deposition. However, anthraquinones can also be biosynthesized in plants. In this work, we report on a sample-driven and GC-MS/MS-based analytical strategy to differentiate between endogenous and exogenous anthraquinones in dried walnut (*Juglans regia*) leaves. Anthraquinone and seven of its derivatives were quantified in 9 dried and 128 fresh samples of leaves from walnut and other deciduous trees from three different countries and nine sampling sites. The drying of walnut leaves in a hot-air electric oven eliminated 80% of anthraquinone concentration. Among the fresh walnut leaf samples, 94% exceeded the 0.01 mg/kg maximum residue limit of anthraquinone, with values up to 0.3 mg/kg. Most derivatives were also present above 0.01 mg/kg. However, in the leaves from other deciduous trees, the compounds were much lower than 0.01 mg/kg. We conclude that the low anthraquinone base levels in most samples may result from atmospheric pollution, whereas the higher anthraquinone residues in walnut leaves likely have an endogenous origin.

## Introduction

From a (bio)chemical and biological perspective, the main identified hazards related to food safety can be divided into three categories.^1^ Biological pollution arises from infestation through bacteria, viruses, protozoa, worms or helminths, and fungi.^2^ Alternatively, food commodities may be toxic either inherently, through endogenous substances of the source organism,^3^ or externally, upon contamination with environmentally produced compounds, like polyaromatic hydrocarbons (PAHs) from, e.g., wildfires.^4^ Third, human activities have also been identified as a food safety hazard after direct contact or secondary contamination with anthropogenic materials, such as pesticides for crop protection,^5^ heavy metals in industrial waste,^6^ or antibiotics from nontherapeutic applications as growth promoters in the cattle industry.^7^ The risk assessment for each substance is a nontrivial estimation that depends on the commodity or matrix being investigated,^5^ among a multitude of other factors. Moreover, some substances are multiple-source compounds, which complicates the hazard identification and characterization phases. Aside from risk assessment, such behavior also negatively influences risk management and the measures taken against the agents. For example, the aforementioned case of PAHs is relevant as environmentally naturally occurring toxins but to a much greater extent as anthropogenic materials from industry or heating systems, since such compounds are generally emitted during the incomplete pyrolysis and combustion of organic matter.^8^ Therefore, if one seeks to control their concentration in, for example, smoked and/or dried products, food processing should not only be carried out without dirty fuels, such as diesel oil, but also avoid long exposure to the ambient air in wide open areas, like in sun drying.^9^

Anthracene-9,10-dione or anthraquinone (AQ) is an oxygenated PAH. As an anthropogenic material, AQ has been implemented in key industrial processes. In the paper and cardboard industry, the pulp yield can be increased with production time reduced through AQ-mediated redox catalysis.^10^ Also, due to its electronic properties, AQ acts as a catalyst in the main industrial process for hydrogen peroxide synthesis with molecular hydrogen and molecular oxygen.^11^ In pest control, AQ has also been implemented in the form of bird-repellent formulations due to its emetic properties upon ingestion causing avoidance conditioning in avian species.^12^ However, its use as pesticide found its legal end in the European Union (EU) in 2009, when the Commission Decision 2008/986/EC entered into force stating that there were clear indications of adverse effects on the environment and human health due to potential carcinogenicity.^13^ In fact, the International Association for Research on Cancer classifies this substance as “possibly carcinogenic to humans (Group 2B)”.^14^ In the same line, the German Federal Institute for Risk Assessment recommended avoiding this substance in food contact materials,^15^ such as paper.

In the 2016 annual pesticide report, the European Food Safety Authority observed that the matrix with the highest frequency of AQ exceedance of the maximum residue limit (MRL) of 0.02 mg/kg in dried products^16^ was tea, mostly from China, with concentrations up to 0.37 mg/kg.^17^ The same European authorities stated back in 2012 that “*[···] the pesticide use of anthraquinone is no longer authorized within the EU [···] and that no uses authorized in third countries were notified [···]*”.^18^ Such an evaluation indicates that AQ residues do not arise from direct contact with tea crop protection products. Moreover, the use of AQ in the paper industry is coming to an end due to technical drawbacks in its implementation with respect to novel processes as well as because of the aforementioned health risk concerns and legislation.^19^ The most probable origin of AQ residues in tea has been linked to its direct emission or to the secondary oxidation of coemitted anthracene during the incomplete pyrolysis or combustion of organic matter for residential heating. Airborne AQ is then adsorbed on tea leaves and eventually absorbed through the stomata.^20,21^ Although contamination during tea processing has also been shown to be significant, this is mainly due to bad practices.^9,20,22^ In fact, when using clean fuels or electrical heating, the drying of leaves can have a positive impact by reducing between 58% and 85% of AQ concentration.^23^

Nevertheless, several plants biosynthesize oxidized AQ derivatives via the polyketide (acetate-malonate)^24^ route or alternatively via a barely used branch of the shikimate pathway (SA).^25,26^ In several *genera* like *Rhamnus*, *Frangula* (both *Rhamnaceae*), *Aloe* (*Asphodelaceae*), *Cassia* (*Fabaceae*), and *Rheum* (*Polygonaceae*), these compounds are known as the main secondary metabolites. Besides, there are numerous reports of AQ occurrence in other *genera* like *Juglans* (*Juglandaceae*) or *Castanea* (*Fagaceae*) synthesizing AQs as minor metabolites besides other polyphenols.^26,27^ According to the literature,^27^ Schwindl et al. recently elucidated the structure of various AQs in the leaves of *Juglans regia*(28,29) and confirmed older reports that walnuts may contain a plethora of AQ derivatives.^26^ As the assessment of AQs and other contaminants by authorities and the establishment of suitable MRLs crucially depend on whether they are endogenous metabolites or exogenous contaminants, an appropriate analytical procedure was developed.

The use of walnut leaves as food or medicinal plants is versatile. In folk medicine, walnut herbal infusion is used for the treatment of eczema and colds, and phytotherapeutically, “*Juglandis folium*” is used externally for mild superficial inflammation of the skin. It is also indicated for hyperhidrosis, *i.e*., excessive sweating, especially on the hands and feet.

**Figure 1:**
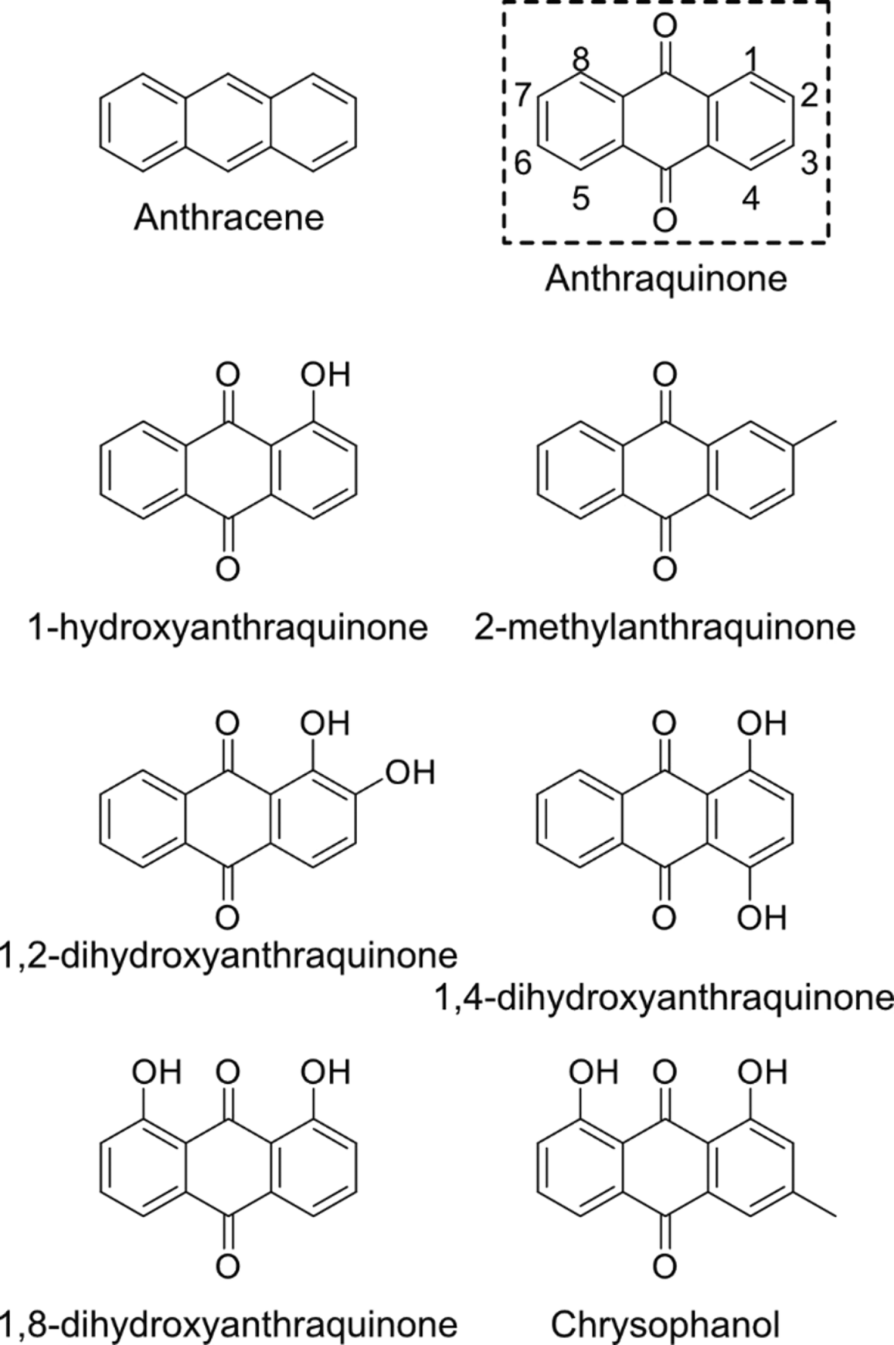
Chemical structure and nomenclature of analyzed anthraquinone and its selected derivatives.

Between 2014 and 2022, 23 samples of hot-air oven-dried leaves from walnuts (*J. regia*) harvested in Bosnia and Herzegovina were analyzed at Labor Friedle GmbH, 100% of which showed AQ residues exceeding the MRL with concentrations ranging from 0.020 to 0.084 mg/kg (data not shown, but available from Labor Friedle GmbH on request). From the scale of the concentration values and the type of matrix, such findings resemble the tea case in the first approximation. To discern the origin of AQ residues in the dried samples, AQ and seven derivatives, anthracene, 1-hydroxy-anthraquinone (1HA), 2-methylanthraquinone (2MA), 1,2-dihydroxy-anthraquinone (12DHA), 1,4-dihydroxy-anthraquinone (14DHA), 1,8-dihydroxy-anthraquinone (18DHA), and chrysophanol (Figure 1), were quantified. The analytes were selected based on a comprehensive bibliographic research, frequency of occurrence, commercial availability of the corresponding analytical standards, and capacity to be quantified with acceptable validated results. The influence of convective drying was investigated with an electric oven under different sets of conditions. Parallelly, a comprehensive sampling program of fresh samples was designed. It entailed 161 fresh samples in total from the countries of Germany and Spain as well as Bosnia and Herzegovina from nine locations, which were investigated in spatially or temporarily resolved replicates of leaves, branches, and the nut parts from *J. regia* specimens, other adjacent deciduous trees, and mosses.

## Materials and Methods

### Sampling

To avoid any systematic bias between trees, dates, and locations, the sampling procedure was always carried out in the same manner. First, six points randomly distributed around the treetop were selected, from which the increments were later collected. With the available sampling material and/or the reduced tree height, 30% or higher treetop coverage could be guaranteed. Second, at each selected point, a branch was either cut or sawed and allowed to fall onto the ground. To each location, a type of replicate sampling strategy was assigned: when information was related to the space dimension, branches and leaves as well as nuts were the materials of choice. For leaves, each increment was treated as a separate sample; for branches and nuts, aggregated samples were made instead. On the other hand, when sampling was carried out in the time dimension, only leaves for an aggregated sample were collected. This distinction was necessary to narrow and limit the research workload. Concerning other deciduous trees, an aggregated sample for each plant and day was made. After these 2 steps, the desired components of the branches were either cut or sawed, separated, and placed on previously analyzed free-of-residue aluminum foils, which were then folded and put into labeled bags. In the case of moss, it was directly sampled with bare hands from the tree trunk and collected on an aluminum foil, which was then folded and put into a labeled bag. Only one moss sample per location was taken due to the small amount present on the trees. To further reduce sample pollution, no sample paper bags were used in this project and all were made from plastic. Moreover, all materials that came in direct contact with the samples were cleaned beforehand and between samples with either acetone, an alcoholic dissolution, or soap. All samples were stored at −20 °C until they were homogenized. Specific information on locations can be consulted in the Supporting Information (SI1, Sampling locations).

### Drying of Leaves

Among the nine total sites, two sampling locations were located in the German villages of Tegernheim and Wiesent, at which every week, a sample of leaves from each corresponding walnut tree was collected. On each field day, ∼60% of the collected samples were separated from the corresponding aggregated sample for each drying experiment, while the other 40% were analyzed fresh, that is, for each oven configuration, two replicates were executed in parallel, one for each location. The samples were dried in the oven Thermo Scientific Heraeus UT 6060 (Waltham), which had previously been configured and turned on to reach equilibrium before starting the drying phase. The drying was carried out until the reference eliminated water percentage of 65–70% was reached, which had been previously determined by Labor Friedle GmbH by heating a fresh leaf sample in the aforementioned oven until no difference in dried sample mass could be observed for longer drying times (data not shown but available from Labor Friedle GmbH on request). After that, the leaves were directly homogenized.

### Sample Comminution

Samples first underwent a cryomilling step with the aid of dried ice in a robot coupe Blixer 3 3.7 L blender (Montceau-les-Mines, France) for 30–60 s to produce coarse particles. The frozen sample was then moved to a Retsch GM 200 grinder (Düsseldorf, Germany) and blended for 3 min at 5000 rpm. The particle size was in the end smaller than 2 mm. Finally, the powder was transferred to a plastic beaker with a lid and stored at −20 °C until the lixiviation. In this way, the exposure of the frozen samples to the laboratory atmosphere was kept under 1 min, thus minimizing air humidity and water deposition.

Nuts required a preprocessing step to separate with a knife and hands the green husks, the shells, and the kernels. From then on, the three parts were considered different samples and analyzed as such. Green husks only needed to pass through the larger blender to achieve the final particle size. On the other hand, the shells required to be shattered into finer pieces with a hammer, before transferring them to the grinder.

In the case of the branches, cryomilling did not enhance the grinding efficiency due to the hardness and the low water content of the matrix. Although a prechopping step with scissors was added to the method, only a particle size smaller than 5 mm could be guaranteed for the final homogenates.

Moss samples were not homogenized according to the aforementioned protocol because the collected mass from the corresponding trees was under 6 g, which made the use of the big blenders unfeasible. Therefore, the ball mill Retsch MM 400 (Düsseldorf, Germany) was implemented instead.

### Analytical Standards and Standard Solutions

Specific commercial information about the acquired analytical standards as well as solvent and concentration of standard solutions can be consulted in the Supporting Information (SI2, analytical standards and standard solutions). All solvents were purchased from Carl Roth (Karlsruhe, Germany).

### Lixiviation

After an extensive optimization phase of different solvent extraction techniques (data not shown), the best-performing method was a modified version of the quick, easy, cheap, effective, rugged, and safe (QuEChERS) protocol.^30,31^ Depending on the density of each matrix, its water content and the available amount for each type of sample, the weighed mass, the added volume of acetonitrile and H_2_O, and the grams of salts were adapted to each case (Table 1). BEKOlut GmbH & Co. KG Citrate-Kit-02 (Bruchmühlbach-Miesau, Germany) prefilled tubes containing 2 g of MgSO_4_, 0.5 g of NaCl, 0.5 g of sodium citrate, and 0.25 g of sodium hydrogen citrate sesquihydrate were used to add the salt mixture. Each vial was allowed to sufficiently increase the activity coefficients of the solutes at a high aqueous ionic strength of up to 5 mL of water.

**Table 1:** Weighed Sample Mass (in g) and Volume (in mL) of Water and Acetonitrile Dispensed to the Test Vials and Number of Salt Mixture Tubes Added to the QuEChERS Extraction Mixture

matrix	sample (g)	H_2_O (mL)	acetonitrile (mL)	no. of salt tubes^a^
dried leaves	2	8	10	2
fresh leaves	5	5	10	2
walnut husks	5	0	5	1
walnut shells	1	9	10	2
walnut kernels	2	8	10	2
branches	1	9	10	2
moss	1	4	5	1

a Tubes prefilled with the salt mixture for the QuEChERS salting-out step. Each vial contained 2 g of MgSO_4_, 0.5 g of NaCl, 0.5 g of sodium citrate, and 0.25 g of sodium hydrogen citrate sesquihydrate.

In the case of dried leaves, fresh leaves, and wood, samples were left for 15 min (30 min in the case of dried leaves) in a Bandelin Sonorex Digitec DT 52 ultrasound bath (Berlin, Germany) before salting out. This step was added to sufficiently soak the samples and better extract the analytes, aided by acoustic cavitation. Additionally, all samples, regardless of the matrix, were left for 3 min in a thermally isolated box filled with dried ice prior to the addition of the salt mixture to reduce the temperature increase due to ion solvation.

### Gas Chromatographic and Mass Spectrometric Separation

All samples underwent gas chromatographic and triple quadrupole mass spectrometric separations for multiple reaction monitoring (MRM) data acquisition. The instruments used were a 7890B gas chromatograph hyphenated with a 7010 mass spectrometer and a 7010B coupled to a 8890, all from Agilent Technologies (Santa Clara).

The carrier gas was He. The inlet was operated in solvent vent mode in all measurements, with 2 μL injected. The initial temperature was 60 °C, which finished after ∼8 s, when a temperature ramp of 700 °C/min started until a final value of 280 °C was maintained for 15 min. The pressure at the inlet and the total flow varied due to retention time (RT) locking after column trimming, but they oscillated around 15 psi and 24 mL/min, respectively. During the split period, which was held for 7 s, the vent outlet had a He flow of 70.0 mL/min and a pressure of 5.8 psi. Between measurements, after the sample injection was completed, the liner was purged for 2 min with a He flow to the vent outlet of 20.0 mL/min. The glass liner was exchanged for a fresh one every ∼30 injections. The oven was set to 50 °C for 1 min. Then, the first ramp of 35 °C/min started, which led to a final temperature of 100 °C after 1.40 min. The next and final ramp had a slope of 8 °C/min, and 27.50 min passed until the final temperature of 320 °C was reached, which was maintained for another 3 min. The run time was in total ∼33 min. The system had a postrun program of 4.1 min, during which the temperature was held at 320 °C and the flow was backflushed to purge the chromatograph. The column used was 30 m long at the beginning of its life, had 0.25 mm internal diameter, and featured 0.25 μm solid-phase thickness composed of dimethylpolysiloxane, 5% polysilarylene-substituted. Two brands were used, which were Agilent Technologies HP-5MS UI and Phenomenex Zebron ZB-SemiVolatiles (Torrance). The column was trimmed 5 cm at the inlet end every 3 days (∼80 injections). RT locking after trimming was conducted using the pesticide chlorpyrifos as a reference substance, since it elutes in the central part of the chromatogram, and a 5-point second-order calibration curve of inlet pressure vs the RT of the reference substance.^32^ The locked RTs of the compounds can be consulted in the Supporting Information (Table S3). 14DHA and 18DHA could not be resolved in this system and were determined together.

Ion generation for mass analysis was carried out for all measurements with an electron impact (EI) ionization source. The EI filament current was 35 μA, and the electrons had an energy of 70 eV. Solvent delay was set at 14 min. A N_2_-filled hexapole was used to produce collision-induced dissociation of the analytes. Mass transition-specific parameters for MRM are listed in the measurement program in the Supporting Information (Table S4).

### Data Analysis

All chromatograms were evaluated using software Agilent MassHunter Workstation Qualitative Analysis for QQQ 10.0 and Agilent MassHunter Workstation Quantitative Analysis for QQQ 10.1 (Santa Clara). Extraction yield, limit of quantification (LOQ), quantification, and drying factor calculations were done in Microsoft Excel 2021 (Albuquerque). Graphs were plotted using both Microsoft Excel 2021 and the programming language Python with the corresponding modules NumPy, Pandas, and Matplotlib as well as its submodule Seaborn. Outliers were determined at a 2-tailed α-risk of 0.05 with standardized residuals, for the linearity measurements, and with Grubb’s test.^33^

As quality control compounds and internal standards to correct for nonspecific errors, the polychlorinated biphenyls (PCBs) #31 and #209 were chosen. PCB-31 and PCB-209 were selected because (a) PCBs are biorthogonal, (b) they partition very well into the organic phase, (c) the former elutes at 17 min and the latter elutes at 26 min, thus controlling the early and the late parts of the chromatograms, and (d) they have been extensively used at Labor Friedle GmbH due to their excellent performance, mostly in QuEChERS applications. These substances were added to all measurements in a concentration of 0.25 ng/μL in the final extract.

The instrument signals used in this work are the area counts of the chromatographic peaks. The complete theoretical framework behind all equations can be found in the Supporting Information (SI4, equations for analytical parameters).

For quantification, the dirty extracts in terms of matrix coextractives made unfeasible the use of acetonitrile external standards due to strong matrix effects, usually causing analyte signal enhancement in the extracts. To overcome this issue, the so-called “matrix-matched standards” were used for quantification. Concentration was then calculated through a one-point matrix-matched standard addition. Additionally, three issues need to be dealt with. First, the concentration of the samples was unknown prior to spiking, and some components showed significant differences between samples that could impair the signal of the standard. Second, some analytes, such as 12DHA, showed a strong dependence on the position in the sequence, understanding by sequence the ∼30 injections until liner exchange. Third, the considerable number of samples made it unfeasible to perform a standard addition for each sample. To overcome these issues, several spiked_sample_–sample pairs for standard additions were run in one sequence. The rest of the sequence was then structured following the strategy called “bracketing”, by which between each pair, other five samples were measured. These were quantified through a one-point matrix-matched external calibration. To do so, one pair of spiked_sample_–sample yielded then the standard signal for the three preceding and two following samples. Thus, all extracts were compared to a standard that encountered inlet and column conditions differing by three injections at most. Moreover, if the response of a standard is not reliable, then, the next closest valid standard in the sequence is used. With this method, the number of standard additions is reduced while still maintaining an acceptable performance. Uncertainty calculations were based on the Gaussian uncertainty propagation law. For the coverage factor for the 95% confidence expanded uncertainty, the quantile of the Student’s *t*-distribution at a 2-tailed α-risk of 0.05 and *n* – 2 dof was used. *n* was determined through a weighted sum of the number of replicates used to calculate the extraction yield and method variance, where the weighting factors are the proportions of variance explained.

The drying factor, *D*, for the oven experiments was calculated using the following expression
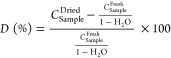
where *C*_Sample_^Dried^ is the concentration of the compound in the dried sample and *C*_Sample_^Fresh^ is the concentration of the compound in the fresh sample. The concentration in the fresh sample was divided by the dried mass remaining after the drying phase, 1 – H_2_O, to account for the matrix reduction and concentration effect. Uncertainty calculation was again based on the Gaussian uncertainty propagation law, using the expanded uncertainties from quantification.

The full analytical method, from the lixiviation step on, was validated for each matrix. Due to the impossibility of finding a dried walnut leaf sample with low concentrations or free of the analytes, a specificity experiment was carried out to find a substitute model leaf matrix for validation. The type chosen was leaves from a tilia tree. The evaluation of the linearity results was done using 0.99 as the lowest value permitted for the squared Pearson’s correlation coefficient. The guidelines proposed by the EU were considered for relative standard deviations of maximum 20%.^34^ The highest permitted LOQ was set at 0.01 mg/kg for all compounds, corresponding to the MRL of AQ in fresh commodities. Complete results can be consulted in the Supporting Information (SI5, matrix-specific method validation).

## Results and Discussion

### Sampling

There are three possible pathways for AQ enrichment in the walnut tree (not considering any manufacturing process such as the drying of leaves), which are (a) through the soil and roots, (b) through the atmosphere and leaves, and (c) through endogenous synthesis. To determine the significance of each route, the sampling strategy was accordingly designed.

Assessing the influence of the soil pathway intrinsically calls for the inclusion of soil and roots in the sampling strategy. However, these were not analyzed in the scope of this work due to the absence of a sampling method safe for the trees. Nonetheless, the significance of such a route is expected to be negligible due to the low translocation factor^35−37^ of such lipophilic substances along the hydrophilic sap-based transport pathways. Leaves are the cornerstone of this work, not only for the atmospheric hypothesis about the adsorption and absorption of AQ but also for the endogenous one. The comparison of the levels found in leaves and other parts of the plant (i.e., nuts and wood) could shed light on this matter: if AQ and its derivatives were to be found in such matrices as well as in leaves, this would suggest that it is the plant that synthesizes these, since transport from other parts of the tree is, as already mentioned, unlikely, and nuts and wood do not possess the high surface and number of stomata of leaves postulated to be the key factor for atmospheric contamination in tea plants. It was initially planned to sample the wood from the bark, as well as from the inner trunk and branches. However, in the end, only the latter were collected because the wounds produced on and in the trunk could have damaged the tree’s integrity. To further investigate the endogenous route, the influence of photosynthesis on the AQ content was assessed by sampling on the same day leaves at different points and depths of the same tree. Oppositely, in order to gain more information on the atmospheric fate of AQ, samples of moss (without taxonomic identification) as a passive sampler growing on the walnut trees were collected following the principle established by Romanotto et al.^20^

Another parameter to consider is the influence of the climatic conditions. This consideration is important not only for the atmospheric hypothesis, since weather can impact the concentration and deposition of airborne chemicals,^21,38,39^ but also for the biosynthesis of secondary metabolites, for it could be promoted as a defense mechanism.^40^ To assess the amplitude of this variable, walnut trees in three countries were selected and the same plants were repeatedly sampled in equidistant time intervals. The weather parameters in the 7 days prior to sampling, such as minimum and maximum temperature, precipitation, wind speed, and pressure, were obtained from the nearest weather station through the Meteostat database.^41^ For temperature, wind speed, and pressure, the mean was computed; for precipitation, the sum was calculated instead.

To assess the influence of anthropogenic factors such as heating systems and industries, the sampling locations were selected in such a way that urban, industrial, as well as rural and wild areas were represented.

The replicate sampling in time is meaningful not only to assess the influence of the weather variations but also to observe the correlation of leaf growth both with the atmospheric hypothesis and the biosynthesis of AQs. In that regard, the circumference at breast height (CBH) as the age estimator of each *J. regia* tree was also measured.

Previous studies at Labor Friedle GmbH had shown that the leaves of other deciduous trees showed traces of AQ under 0.01 mg/kg, whereas a *J. regia* tree standing next to these exceeded the legal limit (data not shown but available from Labor Friedle GmbH on request). Therefore, at each location and on each sampling day, leaves from other deciduous trees in an area of 30 m radius from a walnut plant were collected. The eight selected species were *Alnus glutinosa* (black alder), *Cydonia oblonga* (quince), *Malus domestica* (apple), *Tilia × europaea* (tilia), *Prunus persica* (peach), *Dillenia alata* (red beech), *Fagus sylvatica* (beech), and *Prunus domestica* subsp. *insititia* (damson). For the chosen tree species, there is no evidence in the literature that anthraquinone biosynthesis takes place, and thus, these samples can serve as a blank.

In this strategy, the quantity of 6 increments per aggregated sample represents a compromise between extensive and representative sampling of the statistical population (treetop) and scarce and deficient procedures. This quantity is sometimes called the “magic number” because the confidence interval for the mean decreases drastically with the considered number of increments of the population, until reaching the quantity of six.^42^

### Drying of Walnut Leaves

**Figure 2:**
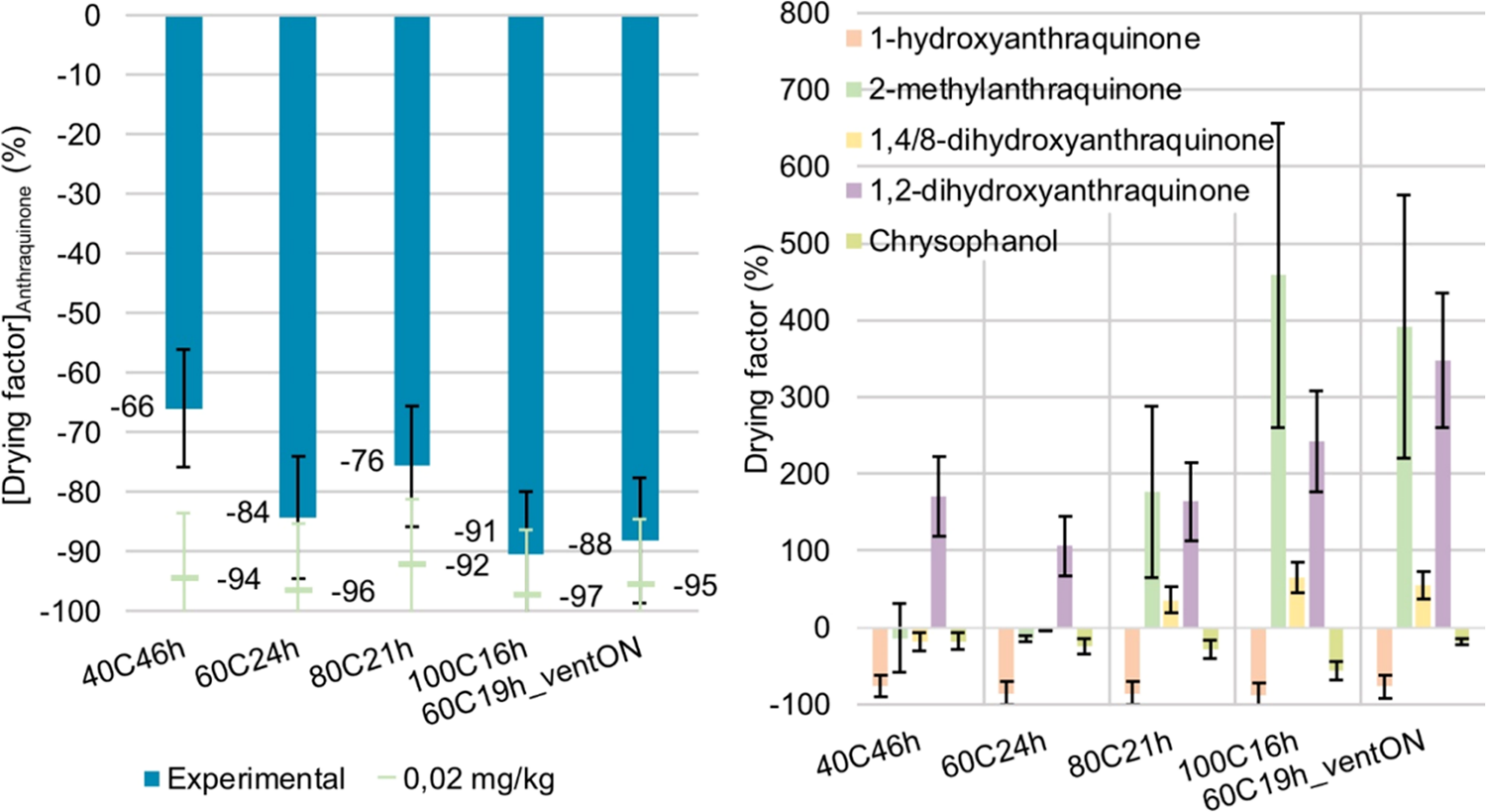
Drying factor mean results of the hot-air oven-drying experiments with the sets of conditions being 46 h at 40 °C without recirculating air (40C46h), 24 h at 60 °C without recirculating air (60C24h), 21 h at 80 °C without recirculating air (80C21h), 16 h at 100 °C without recirculating air (100C16h), and 9h at 60 °C with recirculating air (60C19h_ventON). On the left, the anthraquinone data (experimental) with the theoretical anthraquinone drying factor needed to reach the MRL of 0.02 mg/kg colored in green are shown. On the right, the experimental drying factor of the derivatives is shown. Anthracene was not detected in any of the samples.

Hot-air drying of *J. regia* leaves in an electric oven has a positive impact on the AQ content, regardless of the conditions chosen (Figure 2). Moreover, the drying factor remains with 95% confidence constant at ∼80% of eliminated AQ with respect to the fresh sample, reaching the percentage needed for the legal limit at 0.02 mg/kg throughout all settings except at 40 °C. Since AQ’s vapor pressure is 0.79 Pa at 120 °C,^43^ evaporation during heating can be ruled out. On the other hand, it is unlikely that an enzymatic AQ elimination mechanism is predominant at high temperatures, at which enzymes are usually thermally denatured. A plausible explanation for the disappearance of AQ upon heating could then be a chemical reaction, the specifics of which remain unclear.

To gain more information about the processes occurring to AQ during heating, the drying factors for the other derivatives are evaluated (Figure 2). Overall, 1HA shows the same behavior as AQ, being eliminated regardless of the conditions at a constant drying factor. Although no comparable vapor pressure data to those of AQ could be found, 1HA can be expected to be equally or less volatile than AQ due to its later elution. In consequence, a possible evaporation during drying is also unlikely. Chrysophanol was also eliminated throughout all conditions. However, a certain correlation of the drying factor with the temperature can be observed. Such behavior agrees with nonenzymatic chemical reaction kinetics. A different behavior is shown by 2MA, 148DHA, and 12DHA. Although at conditions milder than 80 °C 2MA seemed to undergo no changes in concentration, it is clear that 2MA was being produced with higher yields the higher the temperature, consistent with a nonenzymatic chemical reaction. 148DHA shows a similar behavior, in the sense that it was produced with increasing temperature and its drying factor remained at 0 below 80 °C. However, it differs on the much smaller scale. Lastly, 12DHA does not show the flipping behavior at 80 °C of 2MA and 18DHA but rather is detected at higher amounts in dried leaves throughout all heating conditions. The temperature dependence at temperatures higher than 60 °C seems to be like its isomer’s. Therefore, a chemical reaction is again the most probable cause.

On average, 1.97 ± 0.76 μmol/kg AQ, 95 ± 26 μmol/kg 1HA, and 0.085 ± 0.078 μmol/kg chrysophanol were eliminated, whereas 0.47 ± 0.21 μmol/kg other analytes were produced. If one hypothesizes that the disappearing mechanism is related to the derivatives’ production during heating, it would be expected that the yield for these compounds remained constant, since predominantly, AQ and 1HA that show a stable drying factor are being eliminated. This suggests that the disappearing compounds are not the primary precursors. On the other hand, such observations might indicate that there is an interplay with a whole other range of compounds not analyzed in this work. The possible reactions seem to be of different nature depending on the compound because the analytes, except AQ, 1HA, and chrysophanol, show different values at 60 °C with and without ventilation and are synthesized during heating. Nevertheless, it can be stated that hot-air drying of *J. regia* leaves in an electric oven clearly has a positive effect on the AQ content. This means that the high residue values found in this matrix are originating earlier.

### Fresh Samples

**Figure 3:**
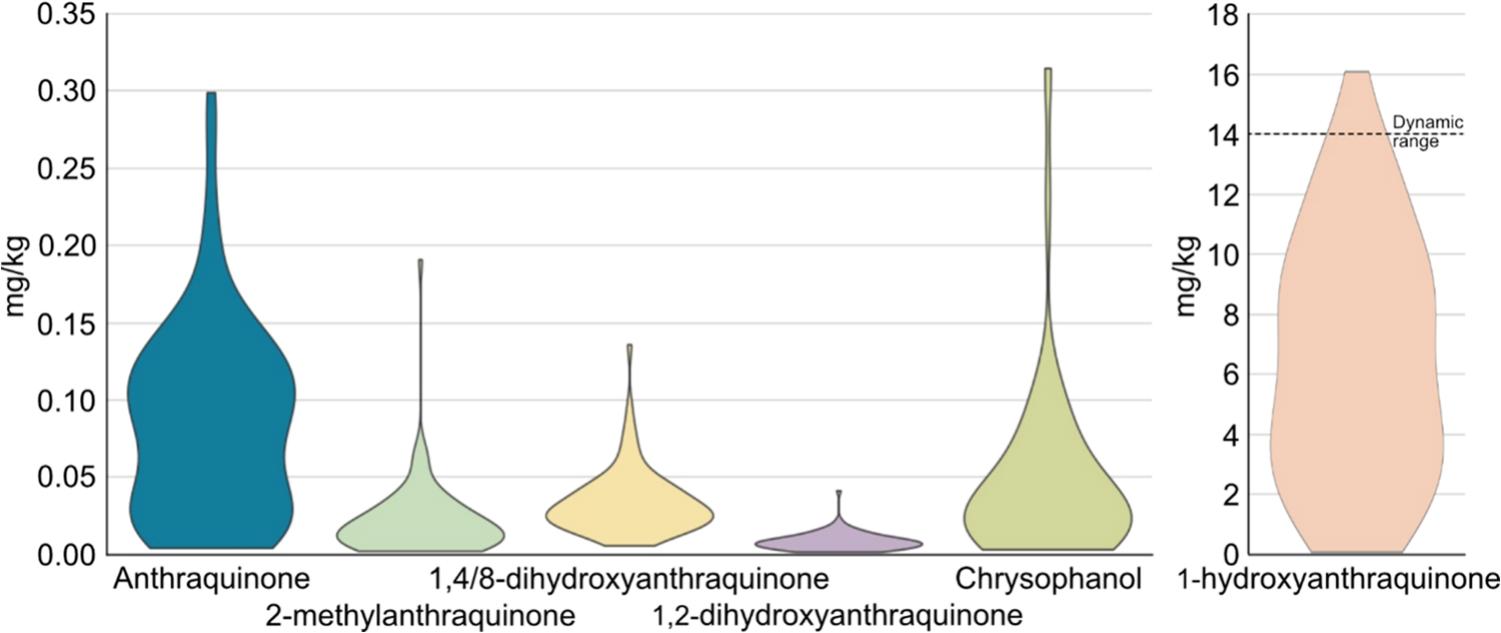
Violin plot for each analyte showing their concentration distributions (in mg/kg) in *J. regia* leaves.

In total, 161 fresh samples were analyzed to gain insights into AQ residues in *J. regia* leaves: 84 walnut leaves, 44 deciduous tree leaves of 8 different species, 8 walnut green husks, 8 walnut shells, 8 walnut kernels, 4 walnut branches, and 5 mosses. Among the *J. regia* leaf samples, 94% of the *J. regia* leaf samples exceeded the 0.01 mg/kg MRL of AQ with values ranging from 0.01 to 0.3 mg/kg. To put the values of the analytes in perspective, a violin plot for each compound showing their concentration distributions in walnut leaves is presented in Figure 3. All anthraquinones appear at around 0.04 mg/kg, whereas 1HA quantification revealed ∼100 times higher concentrations. Such a difference suggests that either the origin of 1HA is different or the hydroxyderivative plays an important role in a different metabolic pathway due to a compound-specific physiological function. However, specifically, the origins of 1HA and AQ do not seem to be disconnected from one another, since they are the only analytes that do not show a homogeneous distribution but rather exhibit a two-peaked kernel density. The lower AQ’s peak appears at the same level as the other analytes. The other four derivatives are also correlated to each other. The current state of knowledge is that the 1,8-dihydroxy derivatives are produced via the polyketide route and the other compounds are produced via the shikimate pathway.^24−26^ This would mean that in the context of endogenous production in walnuts, SA is favored to produce anthraquinones due to the high content of AQ and 1HA. This assumption is also substantiated by the isolation of numerous AQ/1HA structurally similar naphthoquinones such as juglone,^28,29^ which has been quantified in walnut leaves in concentrations above 50 mg/kg^44^ and has been shown to share several biosynthetic steps with AQs.^45^ 2MA and 12DHA, in principle SA metabolites, were quantified a factor of ∼2 less concentrated than 148DHA and chrysophanol, in theory polyketide metabolites. The reason for the higher concentrations of 18DHA and chrysophanol with respect to 2MA and 12DHA cannot be assessed but may be due to compound-specific physiological functions.

**Figure 4:**
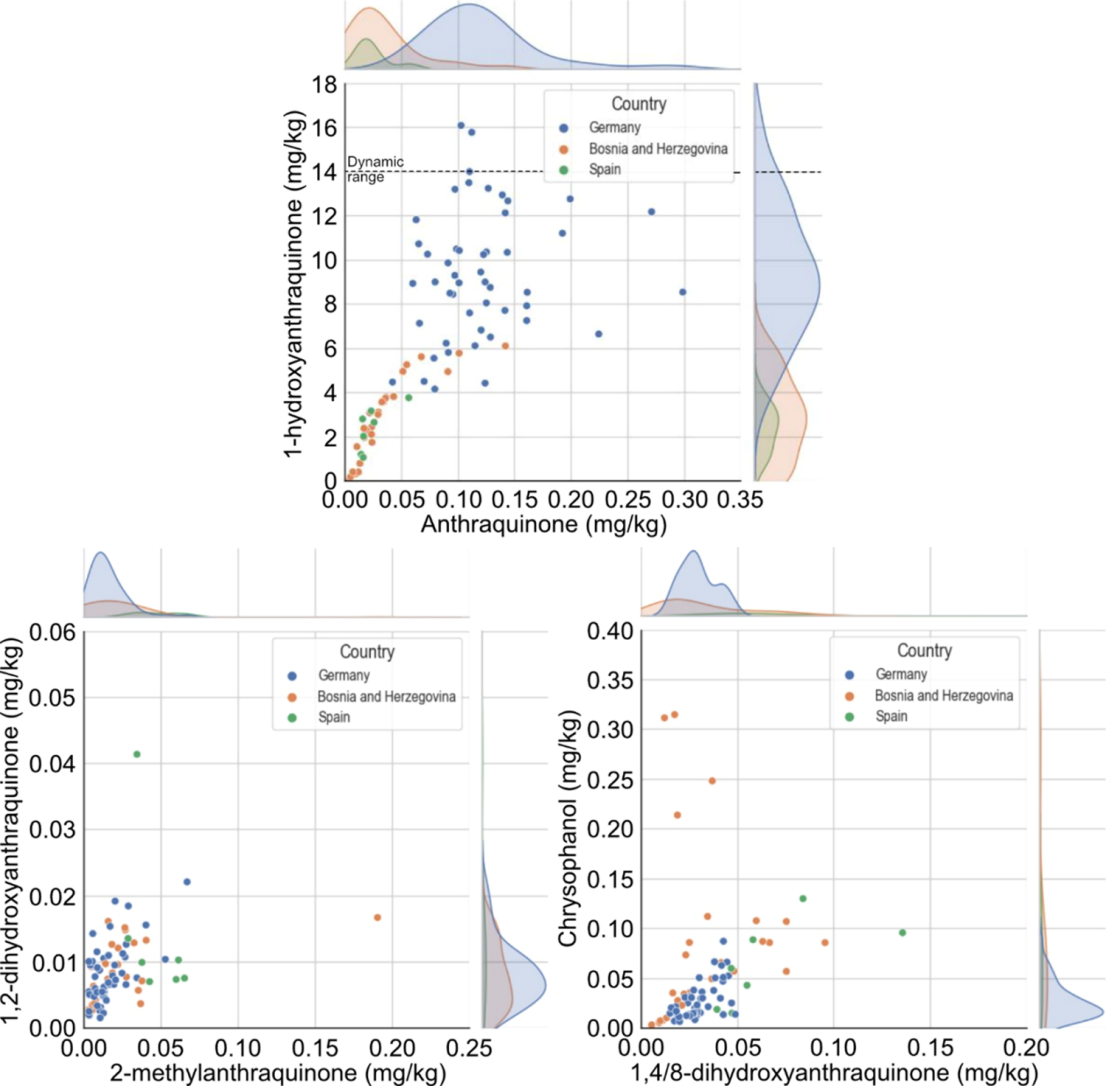
Joint plot of 1-hydroxyanthraquinone vs anthraquinone (top), 1,2-dihydroxyanthraquinone vs 2-methylanthraquinone (bottom left), as well as chrysophanol vs 1,4/8-dihydroxyanthraquinone (bottom right) of concentrations (in mg/kg) in *Juglans regia* leaves, using the semantic variable “Country” as a discriminator.

To test the hypothesis that such differences in distributions may arise from the different sampling locations, a joint plot using the semantic variable “Country” as a discriminator was drawn for AQ vs 1HA, the SA derivatives, and the polyketide metabolites (Figure 4). With these representations alone, a high percentage of the variance in the data can already be visualized. For example, the 2 characteristic peaks of AQ and 1HA are the maxima of the southern countries (i.e., Bosnia and Herzegovina as well as Spain) and Germany. Moreover, the 1HA peak at 4 mg/kg is, in fact, related to the lower AQ level. Another observation that can be made from the AQ-1HA joint plot is that both variables seem to linearly correlate at lower concentrations until approximately 0.05 mg/kg for AQ and 4 mg/kg for 1HA. This linearity further suggests that there may be a metabolic origin of AQ and 1HA, which is not so clear in the German samples. Nevertheless, it is worth mentioning that replicates both in time and space were pooled for this analysis, and the strongest linear correlation can be observed for samples from the same tree, which all happened in Bosnia and Herzegovina (SI7, replicate sampling in space). As expected from the violin plots, the derivatives show no clustering, depending on the country of sampling (Figure 4). This observation also differentiates them from AQ and 1HA. The reason for such behavior could not be assessed. Regarding the linearity of the SA and polyketide metabolites, both pairs show a certain linearity in concentration, which agrees with their biosynthetic origin.

To discern the variables that best fit the variance, the *J. regia* leaf data were modeled via principal component analysis (PCA). Overall, PCA could not clarify the underlying relationships definitively. The main conclusion that can be drawn from it is that precipitation and low temperature seem to have an impact on the AQ concentration. The full discussion can be consulted in the Supporting Information (SI6, principal component analysis).

**Figure 5:**
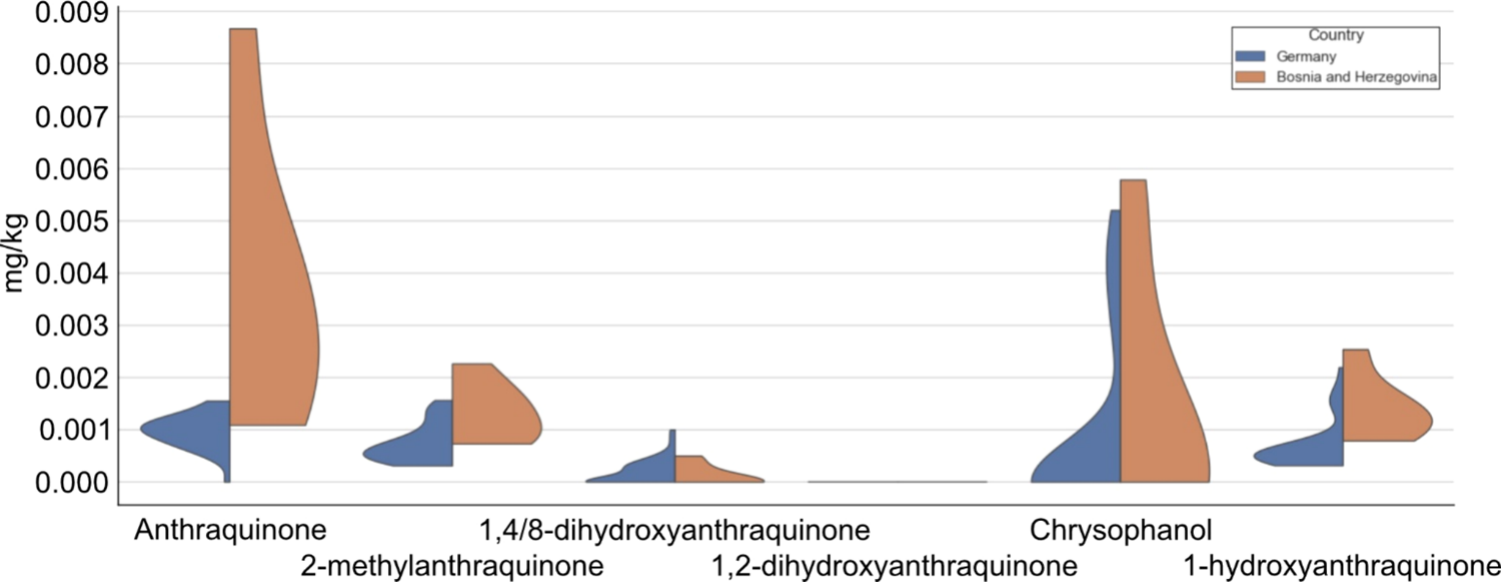
Violin plot for each analyte showing their concentration distribution (in mg/kg) in leaves from deciduous tree different than *J. regia* depending on the country Germany (blue) or Bosnia and Herzegovina (orange).

To assess how important the influence of weather is, the concentrations of the analytes in the other deciduous trees from Bosnia and Herzegovina were compared to those from Germany (Figure 5). German samples do not show higher concentrations of AQ or 1HA with respect to the Bosnians but rather are comparable or even more diluted. This characteristic contradicts the conclusion drawn from PCA and suggests that the weather does not play a decisive role. Another relevant observation is that all values are ∼10 times lower than those in the walnut leaves. Moreover, 1HA exhibits a remarkable reduction of approximately a factor of 1000. This indicates that there is a factor or group of variables different between *J. regia* and other trees. The question arises if such a difference is directly related to the metabolism or rather to the morphology of the walnut leaves that may enhance the uptake from the atmosphere of primarily AQ and 1HA, since they are the most volatile with respect to their retention time. The remarked reduction in the 1HA content speaks against the latter because it is highly unlikely that the morphology of walnut leaves with respect to other trees of eight different species could cause such a significant difference. Muradoğlu and Gündoğdu determined stomata size and frequency in some walnut cultivars.^46^ The determined stomata frequency and size (length and width) were in the range of 183–335 stomata/mm^2^, 17.21–30.10 and 10.65–20.06 (μm), and this is in the normal span of other deciduous trees. Since for the selected tree species, there is no evidence in the literature that anthraquinone biosynthesis takes place, the low AQ base levels may then result from atmospheric pollution.

In samples different from leaves, the compounds were also detected and quantified (Table 2). However, these appear more diluted than in leaves, whereas 148DHA tends to be a factor of ∼3 more concentrated and chrysophanol and 12DHA remain constant, with respect to walnut leaves. 1HA shows again a remarkable drop. Overall, the main compound in the other parts of the plant is 148DHA, followed by 1HA and chrysophanol. 2MA is around a factor of 10 more diluted with respect to leaves. As already discussed, it seems that the plant can produce AQs through two different biosynthetic branches, the polyketide and the shikimate pathway. As chrysophanol and 18DHA are indicators of the polyketide pathway, whereas AQ, 1HA, and 2MA are representatives of SA, it is likely that some metabolic variables or accumulation parameters changed in the different plant parts. This also agrees with the significant reduction in AQ and 1HA concentrations. However, it seems as though what has changed is not the metabolic pathways but rather the amplitude of each one, since the slope between AQ and 1HA in leaves is approximately still maintained in other parts (Figure S13) and the difference between the SA and polyketide metabolites is much clearer.

**Table 2:**
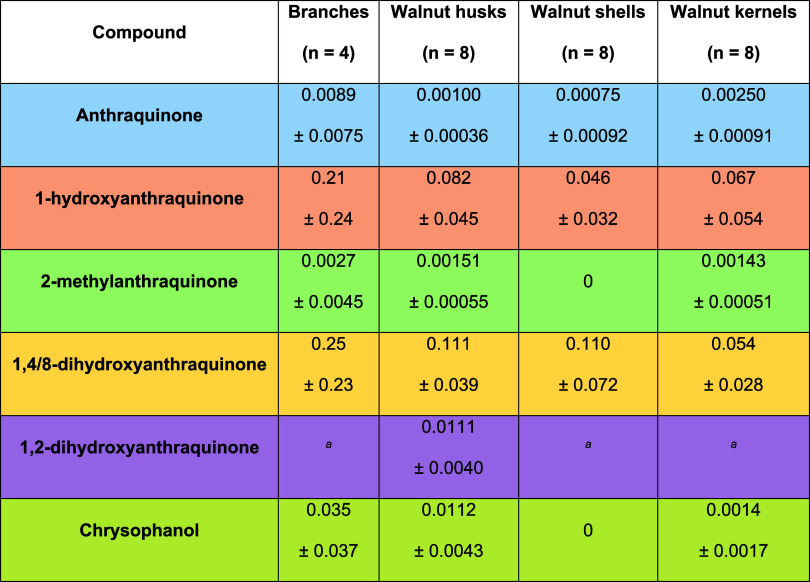
Average Concentration (in mg/kg) of the Analytes in the Different Parts of the Plant, as well as the Expanded Uncertainty at 95% Confidence Level (in mg/kg)

a The analyte did not comply with the required minimum performance set for validation results.

The AQ values in moss (Table 3) fall inside the data distribution of AQ in *J. regia* leaves, which, following the reasoning of Romanotto et al.,^20^ speaks for an atmospheric origin. However, the origin of AQ in moss is not straightforward. On the one hand, the capacity of moss as a passive sampler can be observed with anthracene and 1HA, since these are the samples showing expected traces of this compound. The sampling sites near big cities show concentrated values, whereas the rural Bosnian area in Seona has no traces of anthracene. Accumulation of other AQ derivatives in mosses is not uniform. The presence of all quantifiable compounds is confirmed with a factor of 10 higher compared to anthracene and 1HA but also still in the same range as in walnut leaves. It is unlikely that they come from the tree because the values with respect to branches appear unrelated. As an example, the second main compound 1HA is much more diluted. Nevertheless, it still maintains a linear correlation with AQ, although the slope in moss is in contrast <1 (Figure S14). Therefore, it seems interesting to further analyze the origin of AQs in mosses.

**Table 3:**
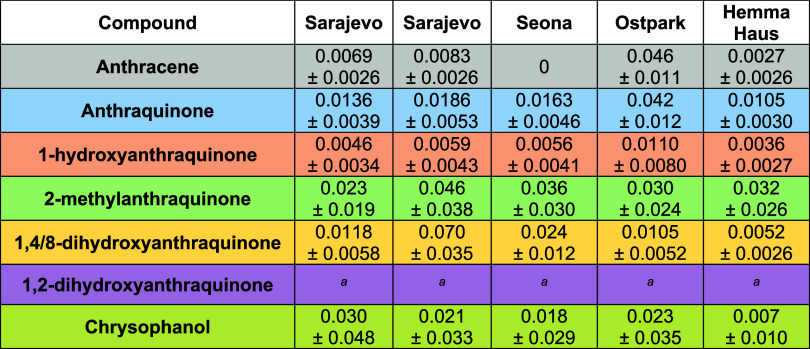
Concentration (in mg/kg) of the Analytes in Moss from Sampling Sites, as well as the Expanded Uncertainty at the 95% Confidence Level (in mg/kg)

a The analyte did not comply with the required minimum performance set for validation results.

To assess the influence of tree age, the CBH measurements can be analyzed. The trees from Germany had on average 71.0 ± 9.0 cm circumference, whereas those from Spain and Bosnia and Herzegovina were 15 ± 1 cm and 100 ± 23 cm, respectively. This indicates that the age of the tree is not the determining factor. One further variable to consider is the influence that light hours could have on the AQ content. To test this hypothesis, the samples taken with replicates in space from Bosnia were analyzed (complete discussion in SI7, replicate sampling in space). From a visual analysis, no correlation between positions located higher as well as on the surface of the treetop and the concentration of the analytes can be determined. This is, however, not a definitive answer because treetops were relatively small and not dense enough to show significant gradients in radiation.

Based on these observations, we conclude that the origin of anthraquinone residues found in oven-dried *J. regia* leaves is predominantly endogenous. The accumulation is likely fed by biosynthesis via the shikimate pathway underlined by 1HA and the particularly rich quinone biochemistry in the family of *Juglandaceae*, which would explain the difference from other deciduous trees. On the other hand, also, compounds like chrysophanol and 18DHA were accumulated to a lower extent, pointing to the biosynthesis via the polyketide pathway for selected metabolites. The reason behind the clustering observed for the German samples with respect to AQ and 1HA remains unclear. The low AQ base levels in other deciduous trees can be linked to atmospheric pollution because there is no literature supporting AQ biosynthesis in the selected specimens. QuEChERS for sample preparation and GC-MS/MS for measurement were found to be versatile tools to quantify several anthraquinone derivatives in parallel with effective LOQs and acceptable validation parameters. Sampling containing walnuts leaves and other plant parts together with other deciduous trees and mosses from nine sampling sites from three different countries allowed observation of various accumulation aspects.

## Supporting Information Available

Details of the sampling sites, analytical standards and standard solutions, instrument measurement parameters, formulas for the calculus of extraction yield, matrix effects, recovery, limit of detection, and limit of quantification, validation results, principal component analysis, and correlation plots of 1-hydroxyanthraquinone vs anthraquinone in different parts of the walnut and moss as well as correlation plots for the replicate sampling in space (PDF)

## Abbreviations

PAH: polyaromatic hydrocarbon
AQ: anthraquinone
EU: European Union
MRL: maximum residue limit
SA: shikimate pathway
*J. regia*: *Juglans regia*
QuEChERS: quick, easy, cheap, effective, rugged, and safe
MRM: multiple reaction monitoring
RT: retention time
12DHA: 1,2-dihydroxyanthraquinone
1HA: 1-hydroxyanthraquinone
2MA: 2-methylanthraquinone
148DHA: 1,4/8-dihydroxyanthraquinone
LOQ: limit of quantification
PCB: polychlorinated biphenyls
CBH: circumference at breast height
PCA: principal component analysis
