# Supplementary material for: Anthraquinone Residues in Dried Walnut (Juglans regia) Leaves for Herbal Infusions: Proof of Endogenous Origin via a Sampling-Driven and GC-MS/MS-Based Strategy
Source: J Agric Food Chem. 2024 Nov 20;72(48):26915–25. doi: 10.1021/acs.jafc.4c08102 (PMC11622234; doi:10.1021/acs.jafc.4c08102)
Supplement: Supplementary file 1 — jf4c08102_si_001.pdf [file jf4c08102_si_001.pdf]

Anthraquinone residues in dried walnut (*Juglans regia*) leaves for herbal infusions – proof of endogenous origin via a sampling driven and GC-MS/MS-based strategy

Lucas Ferrando Plo[1,2]*, Athanasios Nitsopoulos[1], Albrecht Friedle[1,*], Andreas Schmidberger[1], and Jörg Heilmann[2]

[1]*Labor Friedle GmbH, Von-Heyden-Straße 11, 93105 Tegernheim, Germany*

[2]*Institute of Pharmacy, University of Regensburg, Universitätsstraße 31, 93053 Regensburg, Germany*

E-mail: lucas.ferrandoplo@mmk.su.se; afr@labor-friedle.de

SUPPORTING INFORMATION

SI1 – Sampling locations

Banovići (Code: ‘BergLoc2’) 6th of September of 2023

A *J. regia* tree was in the Bosnian mountains near Banovići (Figure 1). Due to the difficult conditions encountered upon arrival, only an aggregated leaf sample was collected. The available material was a telescopic mechanic saw and a pair of scissors. The bags were kept at 4 ºC and sent by post (DHL) after 48 h. The samples arrived five days later at Labor Friedle GmbH. The weather station was placed in Tuzla.

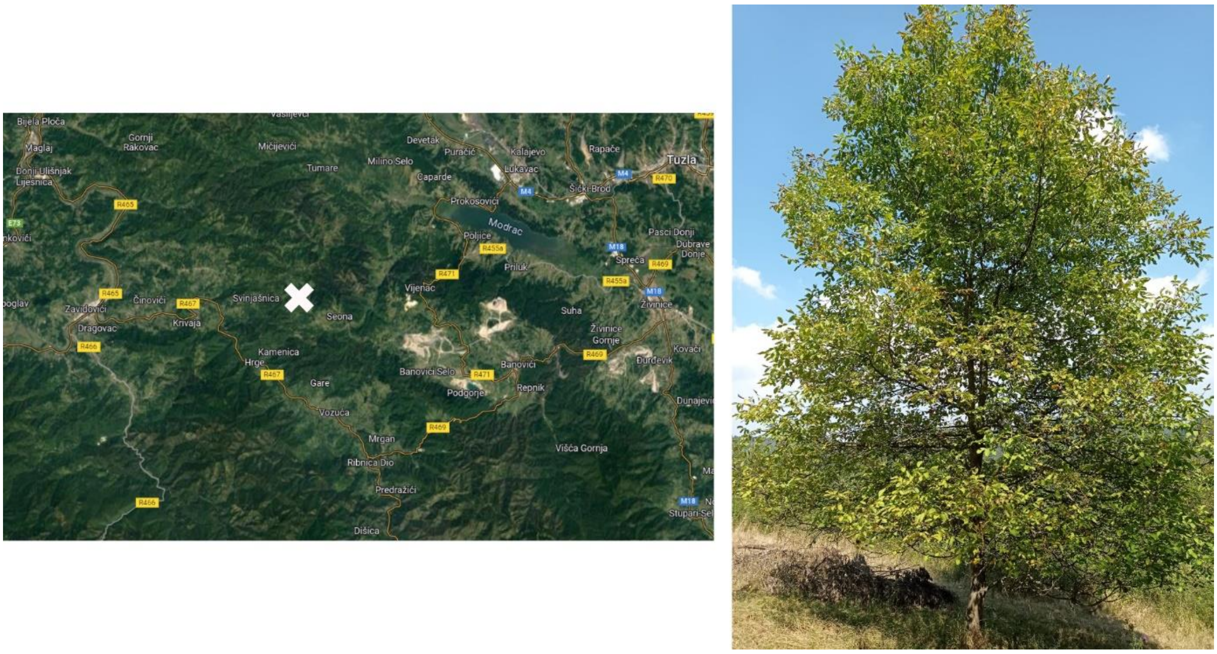

Figure 1. Sampling location in Banovići. Left: location of the selected *J. regia* in the Bosnian mountains, indicated with a white cross; right: selected walnut tree.

Seona (Code: ‘BergLoc1’) 6th of September of 2023

The sampling took place at the village of Seona in the Bosnian mountains (Figure 2). From three walnut trees, which stood next to each other in an area of a radius of 15 m. At the central tree, only five spots were selected, due to the reduced size, and a moss sample was collected. From the other two *J. regia* trees in the area, only an aggregated leaf sample from each plant was taken. Additionally, a black alder nearby was chosen as blank (no photo available). The

available material was a telescopic mechanic saw and a pair of scissors. The bags were kept at 4 ºC and sent by post (DHL) after 48 h. The samples arrived five days later at Labor Friedle GmbH. The weather station was placed in Tuzla.

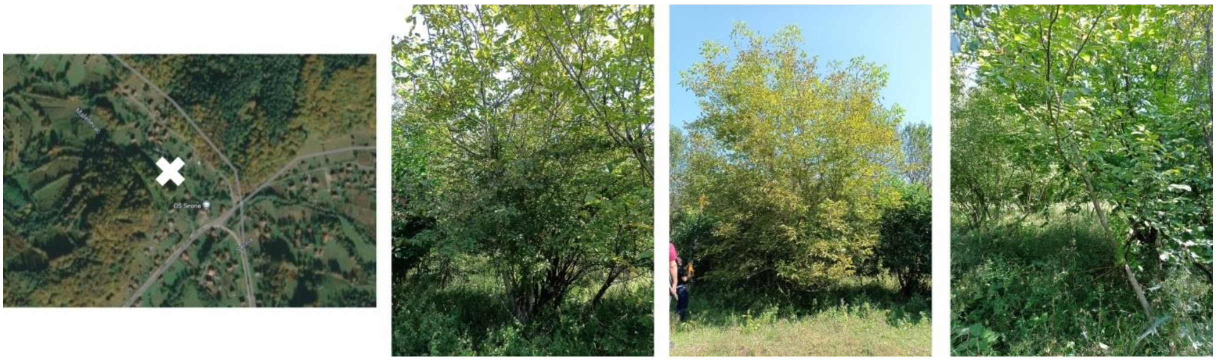

Figure 2. Sampling location in Seona. Left: location of the selected *J. regia* at the village of Seona, indicated with a white cross; right: selected walnut trees, from left to right: ‘central’, ‘area1’ and ‘area2’.

Sarajevo (Code: ‘BB’) 5th of September of 2023

In the city of Binježevo ~10 km away from Sarajevo, five walnut trees were in an area of a radius of 100 m (Figure 3). #2 showed a second very small *J. regia* tree standing 10 m away, from which an aggregated sample of leaves was collected. As blanks, the following trees were chosen: quince for #1 (no photo available), apple for #2, tilia for #3 and peach as well as tilia for #5 (no photos available); #4 had no deciduous tree nearby. Moss was sampled from #1 and #2, separately. Nuts were only present on tree #5, and from #1 and #2 an aggregated sample was made, due to the reduced quantity. The available material was an 8 m ladder, a telescopic mechanic saw and a pair of scissors. The bags were kept at 4 ºC and sent by post (DHL) after 48 h. The samples arrived five days later at Labor Friedle GmbH. The weather station was placed in Sarajevo.

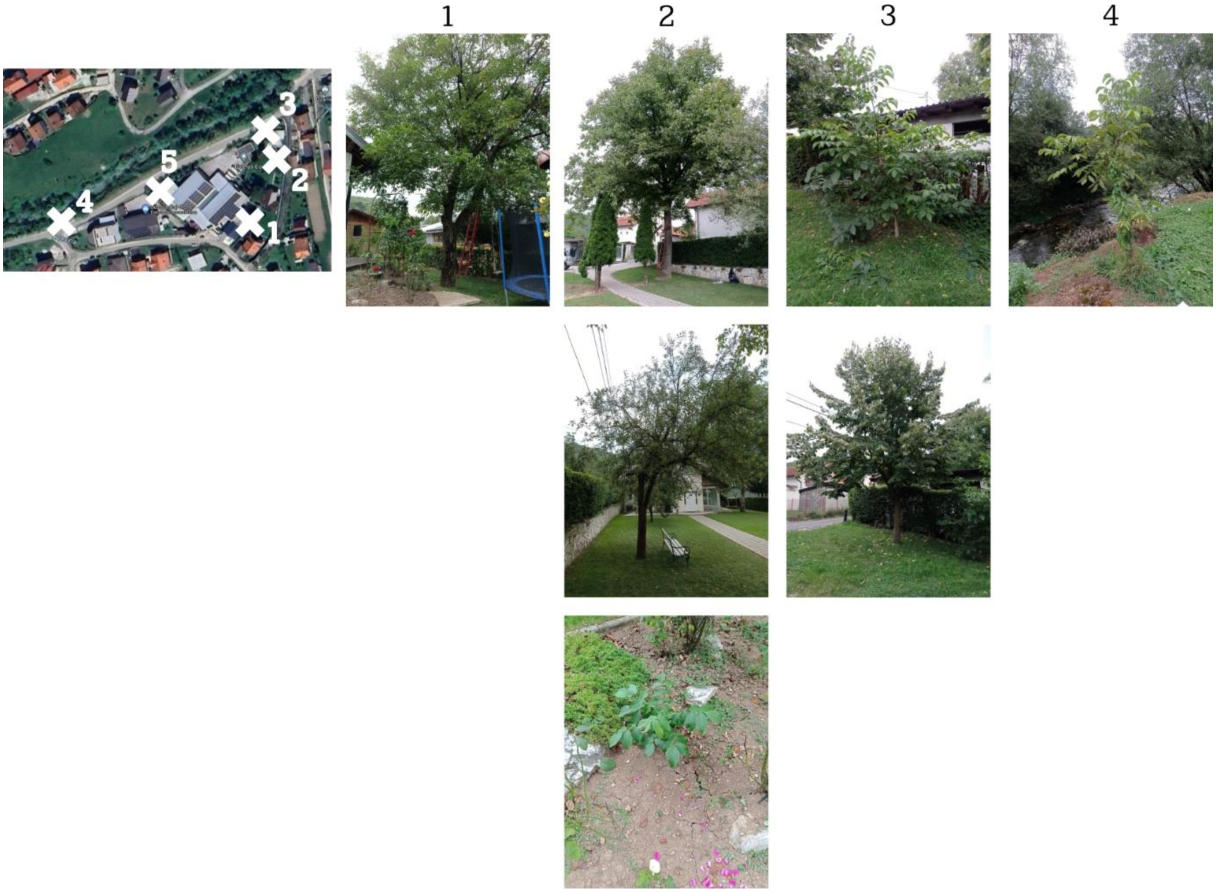

Figure 3. Sampling location in Sarajevo. Left: location of the walnut trees in the city of Binježevo, indicated with white crosses; top right: selected *J. regia* trees (no photo available for #5); middle: deciduous trees, apple tree for #2 and tilia for #3; bottom: small walnut tree, 10 m away from #2.

Caspe (Code: ‘SP’)

The sampling took place at a plantation of 1000 *J. regia* trees of the varieties *Chandler*, *Fernette* and *Franquette* in the region of Caspe in Spain (Figure 4). Six trees were selected, which were sampled by the farmer. At this location, nuts were also periodically sampled. The aggregated samples encompassed all six trees; that is, the population being sampled at this location was not the treetop, but rather the plantation. No blank or moss samples were available at this location. The material was in this case hand-picked and, due to the reduced height of the walnut trees, a 100% treetop coverage was possible. The bags were then sent on the same or following day by post (Correos) and arrived 4 - 5 days later at Labor Friedle GmbH. The sampling period went from the 1st of May to the 18th of September of 2023 and the number of shippings was seven,

which happened every three weeks, on the 1st and 25th of May, the 20th of June, the 10th of July, the 1st and 28th of August and the 18th of September. The weather station was placed in Lérida.

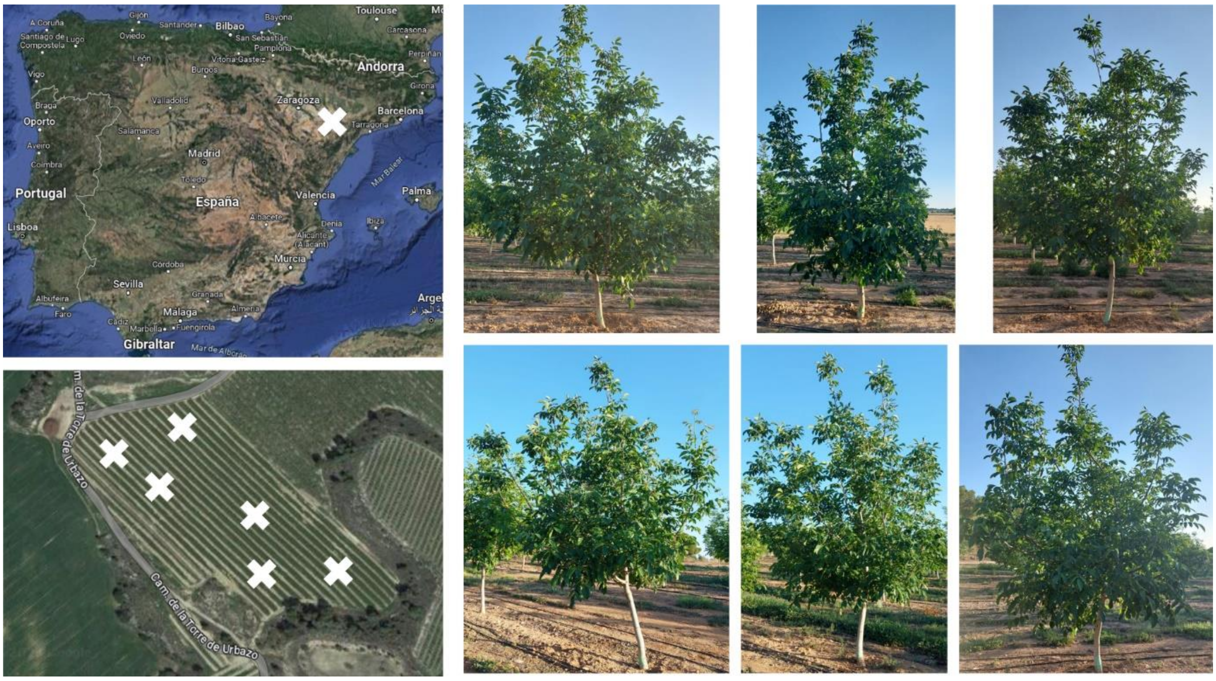

Figure 4. Sampling location in Caspe. Top left: location of the plantation of 1000 *J. regia* trees of the varieties *Chandler*, *Fernette* and *Franquette* in Spain, represented with a white cross. Bottom left: position of the six selected trees in the plantation, indicated with white crosses; right: selected trees.

Haus Hemma (Code: 'Hemma')

A *J. regia* tree was near the city centre of Regensburg (Figure 5), together with a red beech as blank. The available material was a 2 m high ladder and a pair of scissors. The sampling period went from the 15th of June to the 25th of September of 2023, the number of samples for each tree was eight, and they were collected on the same day every two weeks between 17:00 and 19:00, on the 15th and 27th of June, the 13th and 26th of July, the 9th and 24th of August as well as on the 14th and 25th of September. On the last sampling date, a moss sample and an aggregated leaf sample from a very small walnut tree were also collected. The weather station was placed in Regensburg.

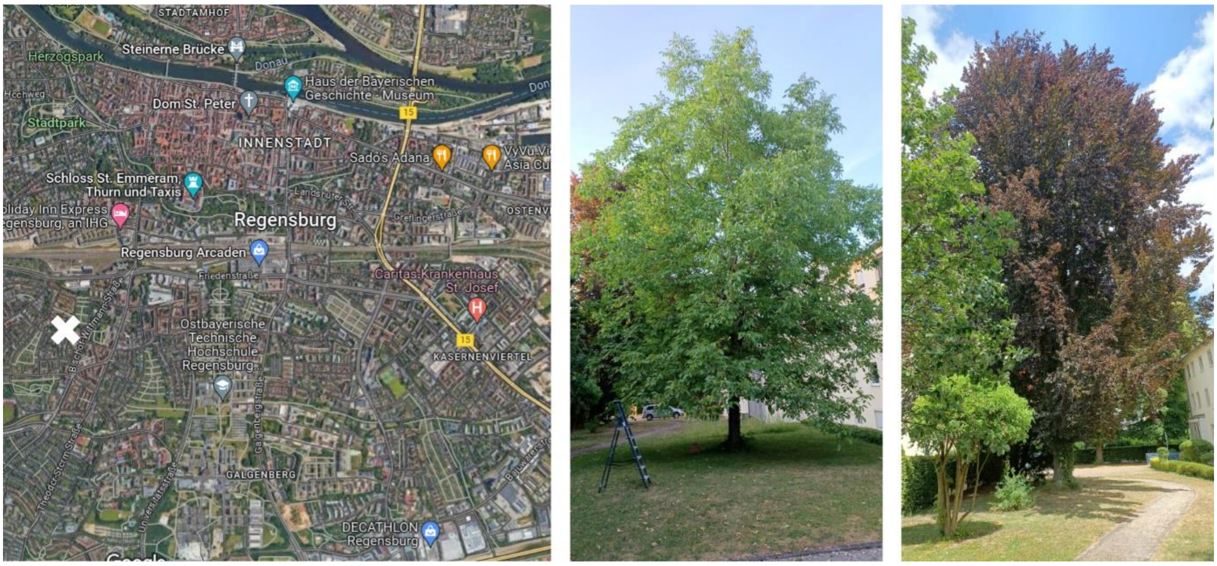

Figure 5. Sampling location in Haus Hemma. Left: location in the city of Regensburg, indicated with a white cross; middle: *J. regia* tree; right: red beech.

Ostpark (Code: 'Ostpark')

At Ostpark in the city of Regensburg (Figure 6), seven *J. regia* trees were located, from which an aggregated sample of leaves for all seven plants was collected; that is, the population being sampled at this location was not the treetop of each tree, but rather the park. Additionally, a tilia tree was selected as blank for this location. The available material was a pair of scissors. The sampling period went from the 16th of June to the 26th of September of 2023, the number of samples for each tree species was eight, and they were taken on the same day every two weeks between 7:30 and 8:30, on the 16th and 27th of June, the 13th and 27th of July, the 8th and 25th of August and on the 15th and 26th of September. On the last sampling day, a moss sample was collected. The weather station was placed in Regensburg.

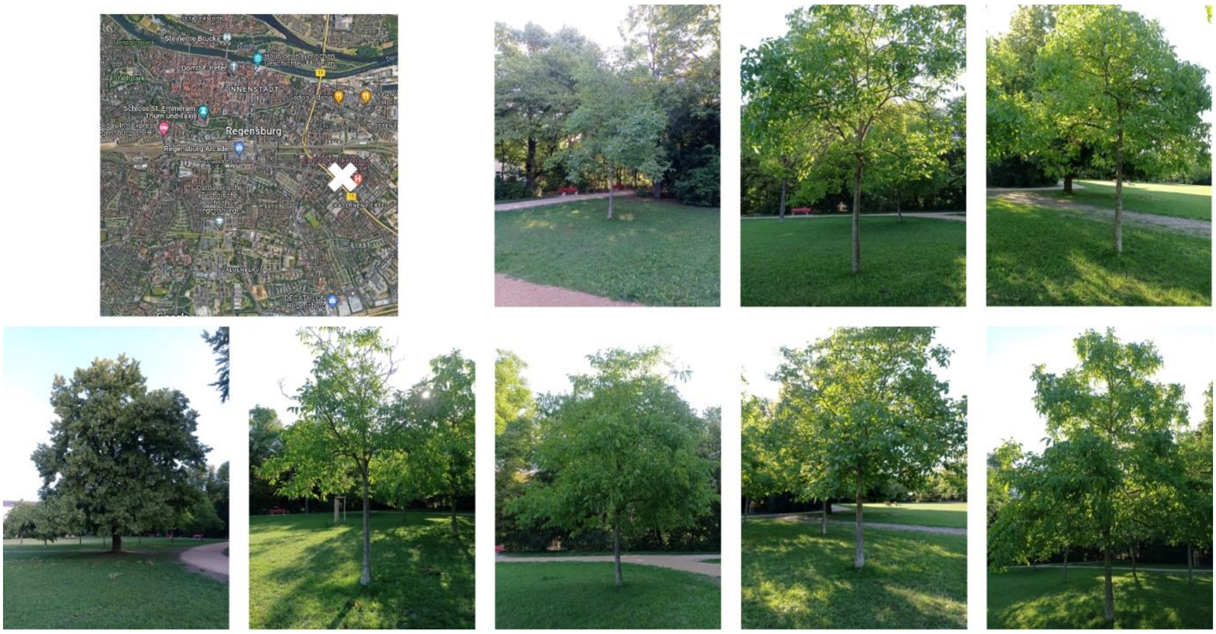

Figure 6. Sampling location in Ostpark. Top left: location in the city of Regensburg, indicated with a white cross; right: selected *Juglans regia* trees; bottom left: tilia tree.

Adlhausen (Code: ‘Ad’)

One km away from the small Bavarian village of Adlhausen (Figure 7), two walnut trees were in a private field. Additionally, a beech was selected as blank for this location. No moss was found on the trees. The sampling was carried out by a worker from Labor Friedle GmbH. The available material was a 6 m long telescopic pair of scissors. The sampling period went from the 21st of June to the 25th of September of 2023 and six samples for each tree species were collected on the same day every two weeks, on the 21st of June, the 6th of July, the 6th and 30th of August and on the 12th and 25th of September. The weather station was placed in Elsendorf-Horneck.

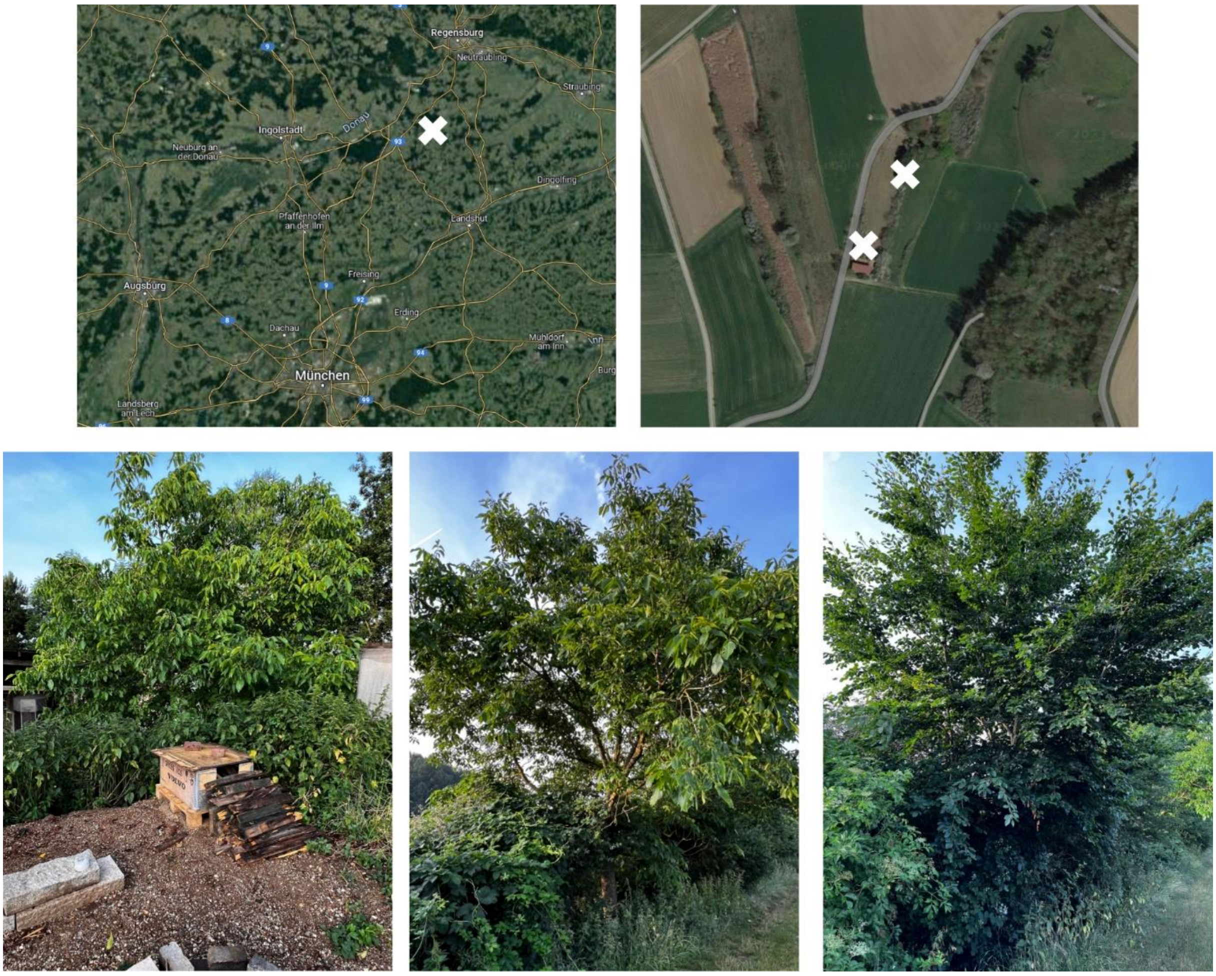

Figure 7. Sample location in Adlhausen. Top left: location of the private field near the Bavarian village of Adlhausen, indicated with a white cross; top right: position of the two selected walnut trees, represented with white crosses; bottom: from left to right, southern *J. regia*, another walnut tree isolated and a beech.

Tegernheim (Code: 'Teg')

In the city of Tegernheim, 30 m away from Labor Friedle GmbH (Figure 8), one *J. regia* tree was in the garden of a private house, together with a damson and an apple tree as blanks. No moss was found at this location. The available material was a 6 m long telescopic pair of scissors. The sampling period went from the 17th of July to 25th of September of 2023, the number of samples for each tree was 10, and they were collected on the same day every Monday between 10:00 and 11:00, on the 17th, 24th and 31st of July; the 7th, 14th and 28th of August as well as the 4th, 11th, 18th and 25th of September. The weather station was placed in Regensburg.

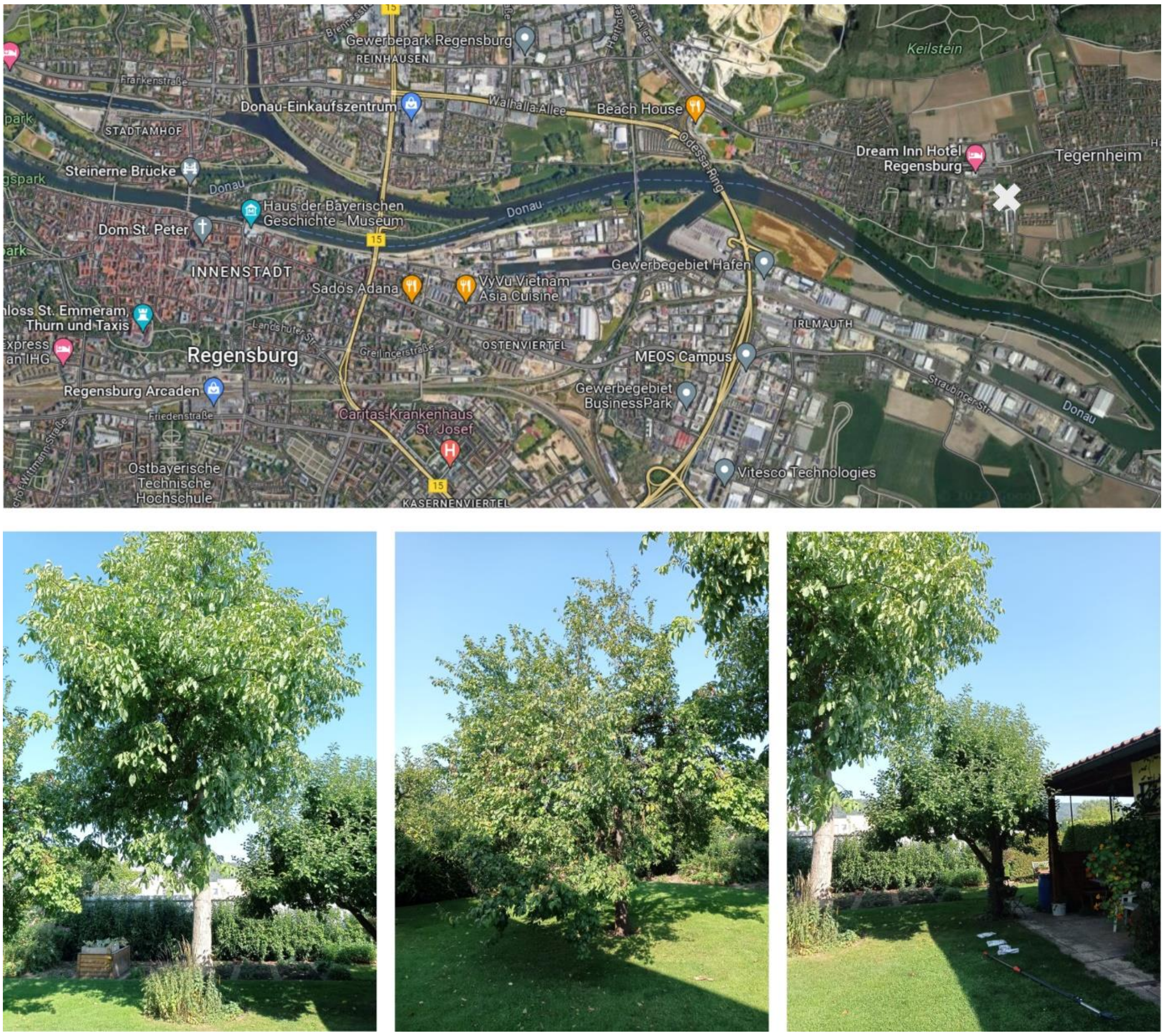

Figure 8. Sampling location in Tegernheim. Top: location of the private backyard 30 m away from Labor Friedle GmbH, represented with a white cross; bottom left: walnut tree; bottom right: from left to right, damson and apple tree.

Wiesent (Code: 'Wi')

In the city of Wiesent, 15 km away from Labor Friedle GmbH (Figure 9), one *J. regia* tree was in the backyard of a private house. No moss or blank tree were available at this location. The walnut tree was sampled by the owner using a pair of scissors. No photos of the tree are available. The sampling period went from the 17th of July to 26th of September of 2023, the number of samples for each tree was 10, and they were collected every Monday between 8:00 and 9:30, on the 17th, 26th and 31st of July; the 7th, 14th and 28th of August as well as the 4th, 11th, 18th and 26th of September. The weather station was placed in Regensburg.

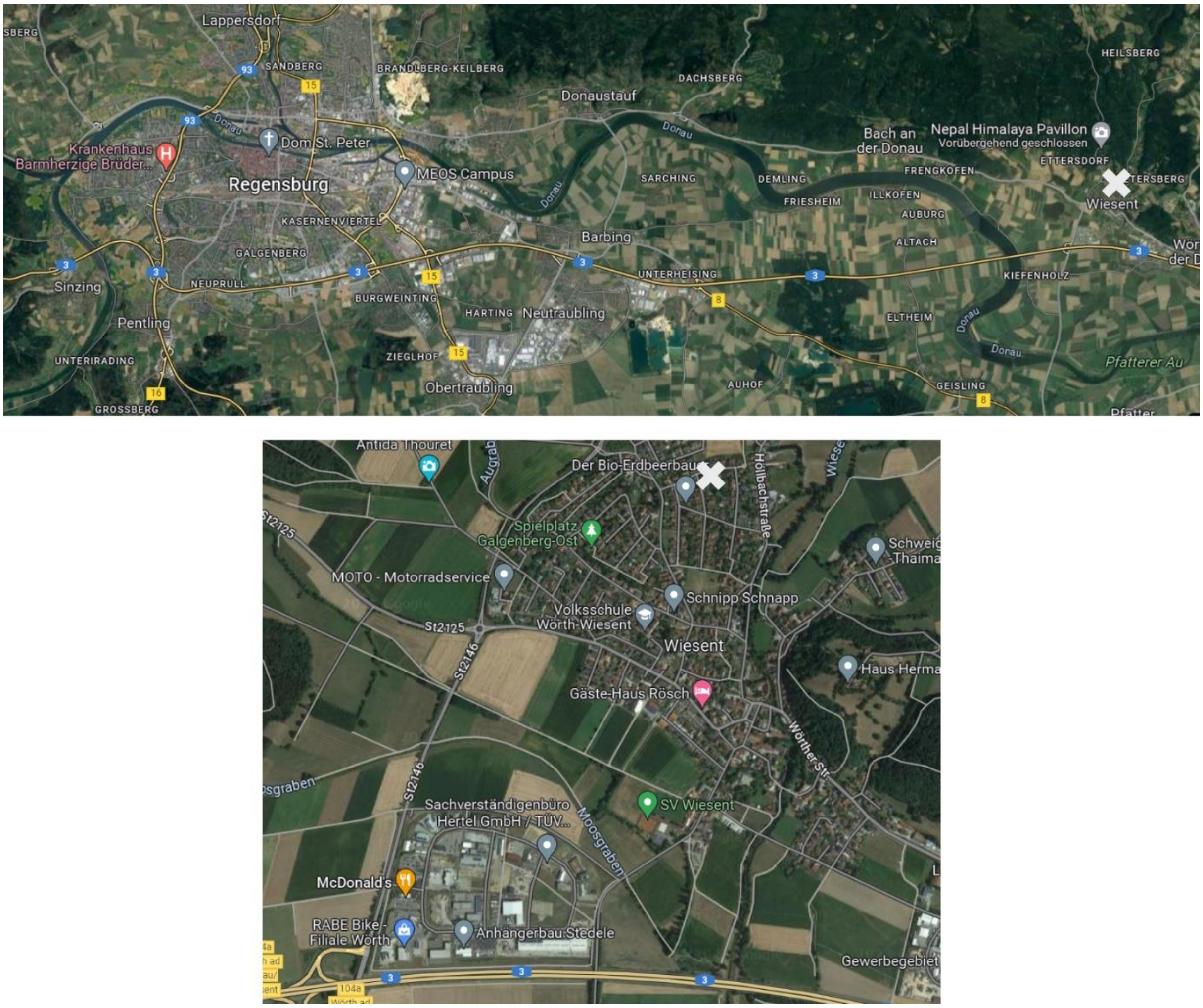

Figure 9. Sampling location in Wiesent. Top: location of the private garden with respect to Regensburg, represented with a white cross; bottom: location of the private house in Wiesent, indicated with a white cross.

SI2 – Analytical standards and standard solutions

Table 1. List of the analytical standards showing, from left to right, the common and IUPAC names, the CAS substance identification number, the batch code, and its purity. PCBs were used as internal standards.

Common nomenclature	IUPAC nomenclature	#CAS	Company	Batch Code	Purity (%)
Anthracene	Anthracene	120-12-7	HPC Standards GmbH	812368	99.12
PCB-31	2,4',5-Trichloro-1,1'-biphenyl	16606-02-3	Dr. Ehrenstorfer GmbH	G1341957	99.47
Anthraquinone	Anthracene-9,10-dione	84-65-1	Fluka	SZBB055Xv	99.80
1-Hydroxyanthraquinone	1-Hydroxyanthracene-9,10-dione	129-43-1	Sigma-Aldrich	CDS000522	99.00
2-Methylanthraquinone	2-Methylanthracene-9,10-dione	84-54-8	Sigma-Aldrich	BCCD0210	99.00
1,4-Dihydroxyanthraquinone	1,4-Dihydroxyanthracene-9,10-dione	81-64-1	Sigma-Aldrich	142002	98.60
1,8-Dihydroxyanthraquinone	1,8-Dihydroxyanthracene-9,10-dione	117-10-2	Sigma-Aldrich	WXBD6572V	95.80
1,2-Dihydroxyanthraquinone	1,2-Dihydroxyanthracene-9,10-dione	72-48-0	CARLO ERBA Reagents GmbH	V2C152112D	98.10
Chrysophanol	1,8-Dihydroxy-3-methylanthracene-9,10-dione	481-74-3	Sigma-Aldrich	BCCG0998	98.30
PCB-209	2,2',3,3',4,4',5,5'-Decachloro-1,1'-biphenyl	2051-24-3	Dr. Ehrenstorfer GmbH	G1057627	98.62

Table 2. List of standard solutions showing, from left to right, the compound name, the solvent used and the concentration (in ng/µL). PCBs were used as internal standards.

Compound name	Solvent	Concentration (ng/µL)
Anthracene	Acetone	200
PCB-31	Cyclohexane	1000
Anthraquinone	Acetone	200
1-Hydroxyanthraquinone	Acetone	1000
2-Methylanthraquinone	Acetone	1000
1,4-Dihydroxyanthraquinone	Acetone	200
1,8-Dihydroxyanthraquinone	Acetone	1000
1,2-Dihydroxyanthraquinone	Acetone	200
Chrysophanol	Acetone	1000
PCB-209	Toluene:hexane 1:1	1000

SI3 – Gas-chromatographic and mass-spectrometric separation

Table 3. Locked retention times (in min) of the compounds investigated. PCBs were used as internal standards.

Compound	Retention time (min)
Anthracene	15.99
PCB-31	16.98
Anthraquinone	18.30
1-Hydroxyanthraquinone	19.58
2-Methylanthraquinone	19.90
1,4-Dihydroxyanthraquinone[a]	20.99
1,8-Dihydroxyanthraquinone[a]	20.99
1,2-Dihydroxyanthraquinone	21.56
Chrysophanol	22.40
PCB-209	27.59

[a] Not resolved neither in the chromatographic nor in the MS/MS dimension.

Table 4. Mass spectrometry parameter list for multiple reaction monitoring, showing (from left to right) the *m/z* value of the precursor and product ions for each compound, the dwell time (in ms) at each transition as well as the corresponding collision induced dissociation energy (in eV). Additionally, the time segments (in min) are presented. The numbers 1, 2, 3 indicate the mass transition of each analyte in order of instrumental response measured with QuEChERS bio pepper extract as analyte protectant. PCBs were used as internal standards.

Compound	Precursor ion (m/z)	Product ion (m/z)	Dwell time (ms)	Collision energy (eV)
14.00 min				
Anthracene_1	178	152	40	30
Anthracene_2	178	128	40	30
Anthracene_3	152	126	40	25
PCB-31	256	186	40	25
Anthraquinone_1	208	180	40	10
Anthraquinone_2	208	152	40	27.5
Anthraquinone_3	180	152	40	15
18.80 min				
1-Hydroxyanthraquinone_1	224	168	40	20
1-Hydroxyanthraquinone_2	224	139	40	30
1-Hydroxyanthraquinone_3	224	196	40	15
2-Methylanthraquinone_1	222	165	40	35
2-Methylanthraquinone_2	222	194	40	10
2-Methylanthraquinone_3	222	166	40	15
20.50 min				

Compound	Precursor ion (m/z)	Product ion (m/z)	Dwell time (ms)	Collision energy (eV)
1,4-Dihydroxyanthraquinone_1[a]	240	128	40	30
1,4-Dihydroxyanthraquinone_2[a]	240	183	40	30
1,4-Dihydroxyanthraquinone_3[a]	184	128	40	15
1,8-Dihydroxyanthraquinone_1[a]	240	184	40	20
1,8-Dihydroxyanthraquinone_2[a]	240	212	40	15
1,8-Dihydroxyanthraquinone_3[a]	212	184	40	10
1,2-Dihydroxyanthraquinone_1	240	184	40	25
1,2-Dihydroxyanthraquinone_2	212	138	40	25
1,2-Dihydroxyanthraquinone_3	184	128	40	15
Chrysophanol_1	254	226	40	15
Chrysophanol_2	254	198	40	20
Chrysophanol_3	226	198	40	10
25.00 min				
PCB-209	498	428	40	25

[a] Not resolved neither in the chromatographic nor in the MS/MS dimension.

SI4 – Equations for analytical parameters

Recovery

To calculate the extraction yield, E, one has to determine the proportion between the signal of a $Spike_{Extraction}$ (*i.e.* extracts obtained spiking before lixiviation) and a $Spike_{GCMS}$ (*i.e.* extracts obtained spiking before gas chromatography). This can be mathematically described by Equation 1:

$$E(\%) = \frac{Spike_{Extraction}}{Spike_{GCMS}} * 100 \qquad (1)$$

where $Spike_{Extraction}$ is the area counts of $Spike_{Extraction}$ and $Spike_{GCMS}$ is the area counts of $Spike_{GCMS}$. In case the sample already contains the analyte, yielding a signal *Sample,* both spike signals need to be corrected by subtracting the sample area counts:

$$E(\%) = \frac{Spike_{Extraction} - Sample}{Spike_{GCMS} - Sample} * 100 \qquad (2)$$

Since internal standards are used in this work to correct for non-specific errors, their signals can be introduced into Equation 2 to obtain values with less RSD in replicability conditions:

$$E(\%) = \frac{\frac{Spike_{Extraction}}{ISTD_{Extraction}} - \frac{Sample}{ISTD_{Sample}}}{\frac{Spike_{GCMS}}{ISTD_{GCMS}} - \frac{Sample}{ISTD_{Sample}}} * 100 \qquad (3)$$

where $ISTD_{Extraction}$ the signal of the internal standard in $Spike_{Extraction}$, $ISTD_{GCMS}$ in $Spike_{GCMS}$ and $ISTD_{Sample}$ in *Sample*. In this work, however, Equation 3 is not valid (read below) and a new approach was needed.

The analytical signal, S, can be explained within the dynamic range of a method as a product of independent factors, f_i, and the concentration of the analyte, C:

$$S = C \prod_i f_i \qquad (4)$$

These factors can be grouped in application-specific and non-specific parameters. The latter, named *Pych*, is related to the physical-chemical law(s) governing signal generation, such as current amplification at the electron multiplier end of a mass spectrometer. The former depend on the sample and the conditions present during its analysis. These were further divided into instrumental factors, *Inst*, like the response that an isolated ion yields at the electron multiplier or the ion transmission factor of the mass analyser, and extraction factors, *Extr*, such as the extraction yield or the matrix effects, *ME*. Equation 4 can then be rewritten considering this clustering:

$$S = C \prod_j Pych_j \prod_k Inst_k \prod_l Extr_l \tag{5}$$

After substituting Equation 5 in Equation 3, one obtains:

$$\frac{\frac{\left(C\prod_j Pych_j \prod_k Inst_k \prod_l Extr_l\right)_{Spike_{Extraction}}}{\left(C\prod_j Pych_j \prod_k Inst_k \prod_l Extr_l\right)_{ISTD_{Extraction}}} - \frac{\left(C\prod_j Pych_j \prod_k Inst_k \prod_l Extr_l\right)_{Sample}}{\left(C\prod_j Pych_j \prod_k Inst_k \prod_l Extr_l\right)_{ISTD_{Sample}}}}{\frac{\left(C\prod_j Pych_j \prod_k Inst_k \prod_l Extr_l\right)_{Spike_{GCMS}}}{\left(C\prod_j Pych_j \prod_k Inst_k \prod_l Extr_l\right)_{ISTD_{GCMS}}} - \frac{\left(C\prod_j Pych_j \prod_k Inst_k \prod_l Extr_l\right)_{Sample}}{\left(C\prod_j Pych_j \prod_k Inst_k \prod_l Extr_l\right)_{ISTD_{Sample}}}}$$

From the definition of the *Pych* factors, it can be derived that these remain strictly constant throughout the measurements. Moreover, the concentration of internal standard is always the same. Additionally, instrumental factors can approximately be regarded as constant since the instruments at Labor Friedle GmbH have undergone extensive validation. Thus, one obtains:

$$\frac{\frac{(C\prod_l Extr_l)_{Spike_{Extraction}}}{(\prod_l Extr_l)_{ISTD_{Extraction}}} - \frac{(C\prod_l Extr_l)_{Sample}}{(\prod_l Extr_l)_{ISTD_{Sample}}}}{\frac{(C\prod_l Extr_l)_{Spike_{GCMS}}}{(\prod_l Extr_l)_{ISTD_{GCMS}}} - \frac{(C\prod_l Extr_l)_{Sample}}{(\prod_l Extr_l)_{ISTD_{Sample}}}}$$

and substituting *Extr* with *E, ME* and *k,* being *k* any other non-analyte-specific variations during extraction, like solvent losses, one obtains:

$$\frac{\frac{(C*E*ME*k)_{Spike_{Extraction}}}{(E*ME*k)_{ISTD_{Extraction}}}-\frac{(C*E*ME*k)_{Sample}}{(C*E*ME*k)_{ISTD_{Sample}}}}{\frac{(C*E*ME*k)_{Spike_{GCMS}}}{(E*ME*k)_{ISTD_{GCMS}}}-\frac{(C*E*ME*k)_{Sample}}{(E*ME*k)_{ISTD_{Sample}}}}$$

Since the k-factors are the same for the analyte and for the internal standard in each spike or sample, and the matrix effects for the analyte and the internal standard are approximately constant for the same matrix and extraction method throughout all measurements, the expression can be simplified to:

$$\frac{\frac{(C*E)_{Spike_{Extraction}}}{E_{ISTD_{Extraction}}}-\frac{(C*E)_{Sample}}{E_{ISTD_{Sample}}}}{\frac{(C*E)_{Spike_{GCMS}}}{E_{ISTD_{GCMS}}}-\frac{(C*E)_{Sample}}{E_{ISTD_{Sample}}}}$$

The addition of the internal standards is always done after the acetonitrile (and water) addition to the test vials, so that their extraction is maximized. Therefore, $E_{ISTD} \approx 1$. Moreover, E_{ISTD} can be regarded as constant for the same matrix and extraction method throughout all measurements due to the extensive implementation of PCBs as quality control for QuEChERS in Labor Friedle GmbH. Additionally, $E_{Spike_{GCMS}} := 1$. The above-derived expression can then be rewritten as:

$$\frac{(C*E)_{Spike_{Extraction}}-(C*E)_{Sample}}{C_{Spike_{GCMS}}-(C*E)_{Sample}}$$

And approximating the extraction of the analyte to a constant for the same matrix and extraction method (which is the case for a validated method):

$$E\,\frac{C_{Spike_{Extraction}}-C_{Sample}}{C_{Spike_{GCMS}}-C_{Sample}E}$$

Since the concentration spiked in $Spike_{Extraction}$ and $Spike_{GCMS}$ are equal, C_{Spike}:

$$E\,\frac{C_{Spike}-C_{Sample}}{C_{Spike}-C_{Sample}E}$$

Therefore:

$$\frac{\frac{Spike_{Extraction}}{ISTD_{Extraction}}-\frac{Sample}{ISTD_{Sample}}}{\frac{Spike_{GCMS}}{ISTD_{GCMS}}-\frac{Sample}{ISTD_{Sample}}}=E\frac{C_{Spike}-C_{Sample}}{C_{Spike}-C_{Sample}E}\neq E$$

Only when the spiked concentration is much higher than the concentration in the sample, $C_{Spike} \gg C_{Sample}$, Equation 3 is true. In this work, all samples showed traces of the analytes and, for some of them, concentration was so high that spiking with high amounts implied working outside the dynamic range. After rearranging the above-presented expression, a more correct equation for the extraction yield is obtained:

$$E(\%)=\frac{C_{Spike}}{C_{Sample}+\frac{C_{Spike}-C_{Sample}}{\frac{\frac{Spike_{Extraction}}{ISTD_{Extraction}}-\frac{Sample}{ISTD_{Sample}}}{\frac{Spike_{GCMS}}{ISTD_{GCMS}}-\frac{Sample}{ISTD_{Sample}}}}}*100 \tag{6}$$

Spike$_{GCMS}$ is fundamentally a dilution of the standard mix with a sample extract, where the dilution factor is determined by the formula $V_{Extract}(\mu L)/100$, $V_{Extract}$ being the total volume (in µL) of the final extract (either 5000 or 10000), and 100 the µL of standard mix pipetted to make a *Spike$_{Extraction}$*. In this work, 200 µL were made for each *Spike$_{GCMS}$*, which means that $200/(V_{Extract}(\mu L)/100) = 20000/V_{Extract}(\mu L)$ of the standard mix and $200 - 20000/V_{Extract}(\mu L)$ of the sample extract were mixed to produce a *Spike$_{GCMS}$*. Since the ISTD in the final extract is then slightly diluted after spiking, one has to introduce the factor H for its signal *ISTD$_{GCMS}$*, such that $H = (200 - 20000/V_{Extract}(\mu L))/200 = 1 - 100/V_{Extract}(\mu L)$:

$$E(\%) = \frac{C_{Spike}}{C_{Sample} + \frac{C_{Spike} - C_{Sample}}{\frac{\frac{Spike_{Extraction}}{ISTD_{Extraction}} - \frac{Sample}{ISTD_{Sample}}}{H\frac{Spike_{GCMS}}{ISTD_{GCMS}} - \frac{Sample}{ISTD_{Sample}}}}} * 100 \quad (7)$$

Since *Sample* and the corresponding *$Spike_{GCMS}$* are done in replicates during validation, the average of these needs to be introduced into Equation 7:

$$E(\%) = \frac{C_{Spike}}{\overline{C_{Sample}} + \frac{C_{Spike} - \overline{C_{Sample}}}{\frac{\frac{Spike_{Extraction}}{ISTD_{Extraction}} - \overline{\frac{Sample}{ISTD_{Sample}}}}{\overline{H\frac{Spike_{GCMS}}{ISTD_{GCMS}} - \frac{Sample}{ISTD_{Sample}}}}}} * 100 \quad (8)$$

Equation 8 gives the extraction yield in percentage for each *$Spike_{Extraction}$* for a given set of extraction conditions.

Matrix effects are the proportion of the signal of a *$Spike_{GCMS}$* with respect to an external standard of same concentration in pure acetonitrile, *ExtStd*:

$$ME(\%) = \frac{Spike_{GCMS}}{ExtStd} * 100 \quad (9)$$

And accounting for the signal from the sample:

$$ME(\%) = \frac{Spike_{GCMS} - Sample}{ExtStd - ACN} * 100 \quad (10)$$

where *ACN* is the signal of the analyte in pure acetonitrile solvent due to contamination and/or impurity. Here no internal standard correction is done because the difference in matrix effects between matrix-rich and pure solvent measurements is not negligible and the ISTD inclusion complicates, rather than simplifies, the final equation. Similarly to the extraction yield, Equation 10 needs to be adapted for the matrices of this work:

$$\frac{(C\prod_j Pych_j \prod_k Inst_k \prod_l Extr_l)_{Spike_{GCMS}} - (C\prod_j Pych_j \prod_k Inst_k \prod_l Extr_l)_{Sample}}{(C\prod_j Pych_j \prod_k Inst_k \prod_l Extr_l)_{ExtStd} - (C\prod_j Pych_j \prod_k Inst_k \prod_l Extr_l)_{ACN}}$$

$$\frac{(C\prod_l Extr_l)_{Spike_{GCMS}} - (C\prod_l Extr_l)_{Sample}}{(C\prod_l Extr_l)_{ExtStd} - (C\prod_l Extr_l)_{ACN}}$$

$$\frac{E(C*ME*k)_{Spike_{GCMS}} - E(C*ME*k)_{Sample}}{(C*E*ME*k)_{ExtStd} - (C*E*ME*k)_{ACN}}$$

In this case, $E := 1$ and $k \approx 1$ for all measurements except for *Sample*:

$$\frac{(C*ME)_{Spike_{GCMS}} - E(C*ME*k)_{Sample}}{(C*ME)_{ExtStd} - (C*ME)_{ACN}}$$

And since the matrix effects in pure solvent are the reference, *i.e.* $ME_{ExtStd/ACN} := 1$:

$$\frac{(C*ME)_{Spike_{GCMS}} - E(C*ME*k)_{Sample}}{C_{ExtStd} - C_{ACN}}$$

Simplifying with $C_{Spike_{GCMS}} = C_{ExtStd}$:

$$\frac{C*ME_{Spike_{GCMS}} - E(C*ME*k)_{Sample}}{C - C_{ACN}}$$

In a validated method, *ME* are constant throughout all matrix-matched measurements:

$$ME\frac{C - E(C*k)_{Sample}}{C - C_{ACN}}$$

So that:

$$\frac{Spike_{GCMS} - Sample}{ExtStd - ACN} = ME\frac{C - E(C*ME*k)_{Sample}}{C - C_{ACN}}$$

After rearranging the expression, one gets:

$$ME = \frac{\frac{Spike_{GCMS} - Sample}{ExtStd - ACN}}{\frac{C - C_{Sample}Ek}{C - C_{ACN}}}$$

If one assumes that the operational errors for sample extraction are under control, then $k \approx 1$:

$$ME(\%) = \frac{\frac{Spike_{GCMS} - Sample}{ExtStd - ACN}}{\frac{C - C_{Sample}E}{C - C_{ACN}}} * 100 \tag{11}$$

Equation 11 gives the matrix effects in percentage for each $Spike_{GCMS}$ for a given set of extraction conditions.

Finally, recovery is the product of extraction yield and matrix effects, which can be obtained with Equation 12:

$$R(\%) = \frac{Spike_{Extraction}}{ExtStd} * 100 \tag{12}$$

With sample subtraction:

$$R(\%) = \frac{Spike_{Extraction} - Sample}{ExtStd - ACN} * 100 \tag{13}$$

Again, no internal standard correction was done due to their intrinsic matrix effects. Following the procedure analogously to the extraction yield and matrix effects, one obtains:

$$\frac{(CC \prod_j Pych_j \prod_k Inst_k \prod_l Extr_l)_{Spike_{Extraction}} - (C \prod_j Pych_j \prod_k Inst_k \prod_l Extr_l)_{Sample}}{(C \prod_j Pych_j \prod_k Inst_k \prod_l Extr_l)_{ExtStd} - (C \prod_j Pych_j \prod_k Inst_k \prod_l Extr_l)_{ACN}}$$

$$\frac{(C \prod_l Extr_l)_{Spike_{Extraction}} - (C \prod_l Extr_l)_{Sample}}{(C \prod_l Extr_l)_{ExtStd} - (C \prod_l Extr_l)_{ACN}}$$

$$\frac{E * ME(C * k)_{Spike_{Extraction}} - E * ME(C * k)_{Sample}}{(C * k)_{ExtStd} - (C * k)_{ACN}}$$

k-factors for measurements in pure solvent are negligible, *i.e.* $k \approx 1$, and for the extracted samples, although they have more sources of error coming from the lixiviation process, they are approximately under control. Therefore:

$$\frac{E * ME * C_{Spike_{Extraction}} - E * ME * C_{Sample}}{C_{ExtStd} - C_{ACN}}$$

Since $C_{Spike_{Extraction}} = C_{ExtStd}$, after rearranging:

$$E * ME \frac{C - C_{Sample}}{C - C_{ACN}} = R * \frac{C - C_{Sample}}{C - C_{ACN}}$$

So that:

$$\frac{Spike_{Extraction} - Sample}{ExtStd - ACN} = R * \frac{C - C_{Sample}}{C - C_{ACN}}$$

which can be expressed as:

$$R = \frac{\frac{Spike_{Extraction} - Sample}{ExtStd - ACN}}{\frac{C - C_{Sample}}{C - C_{ACN}}}$$

Since the samples are extracted and measured in replicate conditions during validation:

$$R(\%) = \frac{\frac{Spike_{Extraction} - \overline{Sample}}{ExtStd - ACN}}{\frac{C - \overline{C_{Sample}}}{C - C_{ACN}}} * 100$$

An additional standard correction factor, *P*, related to the exact dilution of the ISTD in a $Spike_{Extraction}$ due to the volume of ISTD standard mixture, V_{ISTD}, pipetted to the extraction mixture was also added, where $P = V_{Extract}(\mu L)/(V_{Extract}(\mu L) + V_{ISTD}(\mu L))$ as above-explained for the *H* factor.

$$R(\%) = \frac{\frac{P * Spike_{Extraction} - \overline{Sample}}{ExtStd - ACN}}{\frac{C - \overline{C_{Sample}}}{C - C_{ACN}}} * 100 \qquad (14)$$

Equation 14 gives the recovery in percentage for each $Spike_{Extraction}$ for a given set of extraction conditions.

Limit of detection (LOD) and limit of quantification (LOQ)

LOD and LOQ calculations in this work were based on noise evaluation of a sample extract:

$$LOD = \frac{3N}{\frac{S}{C}} \tag{15}$$

where *N* is the half intensity of the baseline core noise of the chromatogram and *S* is the base-line-corrected intensity of a $Spike_{Extraction}$ of concentration *C*. The half height of the noise approximately equals $2\sigma_{Noise}$, where σ_{Noise} is the standard deviation of the gaussian distribution of the noise. The factor 3 yields a signal intensity for the LOD that lies six times the noise standard deviation over the baseline. For the LOQ, the distance is increased to $10\sigma_{Noise}$:

$$LOQ = \frac{5N}{\frac{S}{C}} \tag{16}$$

Quantification

Concentration determination using $Spike_{GCMS}$ and the corresponding *Sample* was calculated using the single-point standard addition method with internal standard correction:

$$C_{Sample} = \frac{\frac{Sample}{ISTD_{Sample}}}{\frac{\frac{Spike_{GCMS}}{ISTD_{GCMS}} - \frac{Sample}{ISTD_{Sample}}}{C}} \tag{17}$$

The numerator corresponds to the intercept of the single-point calibration line and the denominator to the slope. For the same reasons as above explained, an *H* and *P* factors can be included to account for internal standard dilution upon $Spike_{GCMS}$ spiking and standard mix dilution upon $Spike_{Sample}$ spiking, respectively. The extraction yield was also added.

$$C_{Sample} = \frac{\frac{Sample}{ISTD_{Sample}}}{\frac{H * P \frac{Spike_{GCMS}}{ISTD_{GCMS}} - \frac{Sample}{ISTD_{Sample}}}{C}} * \frac{1}{E} \quad (18)$$

In case of anthraquinone (AQ) and 1,4/8-dihydroxyanthraquinone (148DHA), a certain amount of area counts had to be subtracted from the standard signal due to the 0.1% (m/m) impurity of the 1HA standard, which was 40 times more concentrated in leaves for quantification. To determine the amount that had to be deducted, a clean enough extract of tilia leaves was spiked with 0, 2, 6, 10 and 14 (mg/kg) of 1HA. The slope of the correlation between the area counts of 1HA and AQ, m_{AQ}, (Figure 10) as well as 148DHA, m_{148DHA}, was used to determine the area counts to be subtracted from the standards.

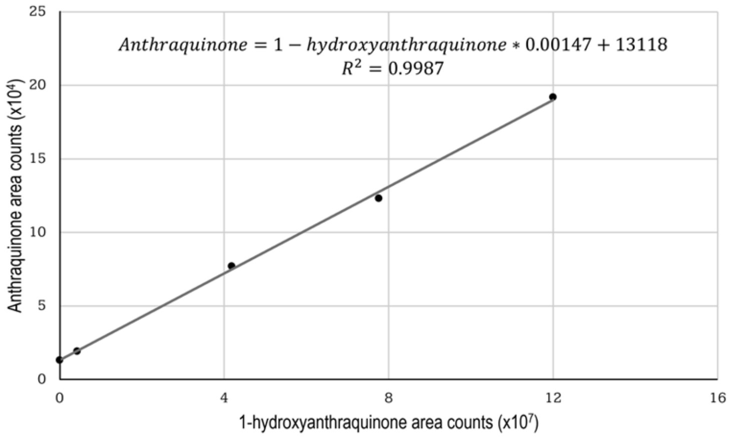

Figure 10. Correlation between area counts of mass transition #1 of 1-hydroxyanthraquinone and #2 of anthraquinone from the spikes of a tilia tree leaf extract at concentrations 0, 2, 6, 10 and 14 (mg/kg) of 1-hydroxyanthraquinone.

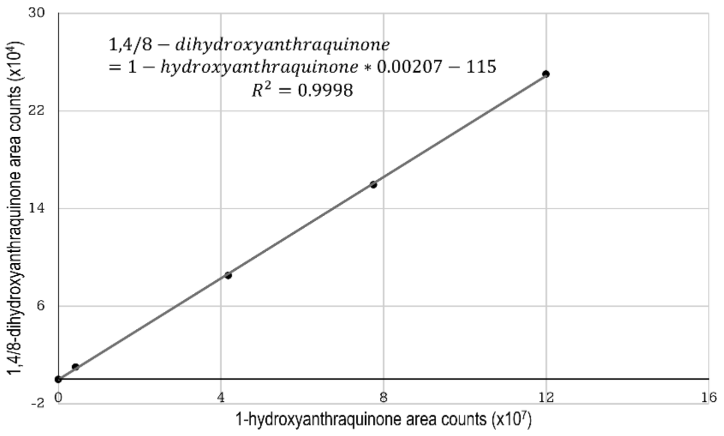

Figure 11. Correlation between area counts of mass transition #1 of 1-hydroxyanthraquinone and #1 of 1,8-dihydroxyanthraquinone from the spikes of a lime tree leaf extract at concentrations 0, 2, 6, 10 and 14 (mg/kg) of 1-hydroxyanthraquinone.

Equation 18 for standard addition is, in the case of AQ and 148DHA, rewritten to Equation 19:

$$C_{Sample} = \frac{\frac{Sample}{ISTD_{Sample}}}{\frac{H * P \frac{Spike_{GCMS} - (1HA - H * 1HA_{Sample}) * m_i}{ISTD_{GCMS}} - \frac{Sample}{ISTD_{Sample}}}{C}} * \frac{1}{E} \quad (19)$$

Where 1HA is the signal of 1-hydroxyanthraquinone in $Spike_{GCMS}$ and $1HA_{Sample}$ is the signal already in the sample.

The other samples, *Sample'*, whose extract was not spiked, were quantified based on a single-point external calibration with a matrix-matched standard. To obtain the area counts of the reference, the signal of its corresponding sample, *Sample*, had to be subtracted.

$$C_{Sample'} = \frac{\frac{Sample'}{ISTD_{Sample'}}}{\frac{H * P \frac{Spike_{GCMS}}{ISTD_{GCMS}} - \frac{Sample}{ISTD_{Sample}}}{C}} * \frac{1}{E} \quad (20)$$

And in the case of AQ and 148DHA:

$$C_{Sample'} = \frac{\frac{Sample'}{ISTD_{Sample'}}}{\frac{H * P \frac{Spike_{GCMS} - (1HA - H * 1HA_{Sample}) * m_i}{ISTD_{GCMS}} - \frac{Sample}{ISTD_{Sample}}}{C}} * \frac{1}{E} \quad (21)$$

Uncertainty calculation was based on the Gaussian uncertainty propagation law:

$$u = \sqrt{(C_{Sample}\frac{u_E}{E})^2 + (C_{Sample}RSD_{Validation})^2 + (C_{Sample}0.01)^2}$$

$$u = C_{Sample}\sqrt{(\frac{u_E}{E})^2 + RSD_{Validation}{}^2 + 0.01^2} \quad (22)$$

Drying factor

$$U = 100D\sqrt{\left(\frac{\sqrt{U^2_{C^{Dried}_{Sample}} + U^2_{\frac{C^{Fresh}_{Sample}}{1-H_2O}}}}{C^{Dried}_{Sample} - \frac{C^{Fresh}_{Sample}}{1-H_2O}}\right)^2 + \left(\frac{U_{\frac{C^{Fresh}_{Sample}}{1-H_2O}}}{\frac{C^{Fresh}_{Sample}}{1-H_2O}}\right)^2} \quad (23)$$

where *U* is the expanded uncertainty at a 95% confidence level from quantification.

SI5 – Matrix-specific method validation

Since dried leaves was the matrix in which anthraquinone residues were initially quantified, a comprehensive validation was planned. Due to the impossibility of finding a dried walnut leaf sample with low concentrations or free of the analytes, a specificity experiment was carried out. Commercially acquired dried leaves and tilia tree as well as red beech leaves oven-dried for 24 h at 80ºC were compared. Red beech and tilia trees were selected for the experiment because they were almost free from analytes and their leaves are morphologically very different with respect to each other and the walnut's. $Spike_{Extraction}$ triplicates for each type were done at concentrations sufficiently high in relation to the values found in the commercial walnut leaves. The spiking levels were anthracene 0.01, AQ 0.05, 1-hydroxyanthraquinone (1HA) 2, 2-methylanthraquinone (2MA) 0.4, 1,48DHA 0.3, 1,2-dihydroxyanthraquinone (12DHA) 0.2 and chrysophanol 0.1 (mg/kg). The sample-corrected $Spike_{Extraction}$ signals (that is, the signal of the sample subtracted to $Spike_{Extraction}$) were used to compute the RSD between the means of each leaf type and the relative differences, Δ_{rel} (*i.e.* the difference of the signal in walnut leaves with respect to each of the other two trees divided by the tilia tree's or the red beech's). Results are shown in Table 5. The method is sufficiently replicable between leaf types, while the lime tree leaves are more proximate to the walnuts. Therefore, leaves from a tilia tree were chosen as model leaf matrix.

Table 5. Specificity experimental results to find a suitable substitute model matrix of dried walnut leaves for method validation with commercially acquired dried walnut leaves and tilia tree as well as red beech leaves oven-dried at 80 ºC for 24 h. The RSD values represent the relative standard deviation between the sample corrected *SpikeExtraction* means of the 3 replicates for each of the 3 types of leaves. The relative difference, Δ_{rel}, with respect to the tilia tree or the red beech dried leaves shows the difference of the signal in dried walnut leaves with respect to each of the other 2 types divided by the tilia tree's or the red beech's. The numbers 1, 2, 3 indicate the mass transition of each analyte.

Compounds<br>Mass transition	RSD (%)	Δ_{rel} (%) dried tilia tree leaves	Δ_{rel} (%) dried red beech leaves
Anthracene_1	6.0	1.5	11.8
Anthracene_2	11.5	2.0	21.7
Anthracene_3[a]			
Anthraquinone_1[a]			
Anthraquinone_2	9.9	17.6	6.8
Anthraquinone_3	11.6	20.8	10.9
1-Hydroxyanthraquinone_1	17.7	12.6	25.1
1-Hydroxyanthraquinone_2	17.1	11.9	24.8
1-Hydroxyanthraquinone_3	18.1	14.3	23.8
2-Methylanthraquinone_1	19.5	21.0	15.6
2-Methylanthraquinone_2	18.4	18.0	18.3
2-Methylanthraquinone_3	20.2	20.9	17.7
1,4-Dihydroxyanthraquinone_1	26.8	[b]	[b]
1,4-Dihydroxyanthraquinone_2	25.9	[b]	[b]
1,4-Dihydroxyanthraquinone_3	26.3	[b]	[b]
1,8-Dihydroxyanthraquinone_1	21.2	0.1	48.7
1,8-Dihydroxyanthraquinone_2	21.3	0.9	49.6
1,8-Dihydroxyanthraquinone_3	20.6	7.8	39.4
1,2-Dihydroxyanthraquinone_1	33.0	10.4	78.9
1,2-Dihydroxyanthraquinone_2	29.4	26.9	31.8
1,2-Dihydroxyanthraquinone_3	32.2	23.6	49.3

Compounds<br><br>Mass transition	RSD (%)	Δ_{rel} (%) dried tilia tree leaves	Δ_{rel} (%) dried red beech leaves
**Chrysophanol_1**	18.4	8.7	44.5
**Chrysophanol_2**	16.5	5.6	30.5
**Chrysophanol_3**	40.4	34.2	138.7

[a] The transition suffered from an unresolvable interference in the three leaf types. [b] The transition suffered from an unresolvable interference in dried walnut leaves.

Method validation for dried leaves (Table 6 and 7) entailed determining the dynamic range in 11 acetonitrile standards at 0, 0.2, 0.4, 0.6, 0.8, 1, 1.2, 1.4, 1.6, 1.8 and 2 (mg/kg) and in $Spike_{Extraction}$ standards with 8 points at the levels 0, 0.005, 0.01, 0.4, 0.8, 1.2, 1.6 and 2 (mg/kg); the recovery as well as the replicability and reproducibility at 0.4 mg/kg and at 0.01 mg/kg, with six replicates of $Spike_{Extraction}$ and three of *Sample*, from which three $Spike_{GCMS}$ replicates were made. By making each $Spike_{GCMS}$ from each sample replicate, instead of the three from the same one, the mean value of $Spike_{GCMS}$ yields a more representative matrix effect estimation. The LOD and LOQ were also calculated. The reproducibility measurements were carried out in two weeks in five separate days and at 2 different instruments, placing the $Spike_{Extraction}$ directly after the routine measurements of Labor Friedle GmbH.

Table 6. Recovery results for the validation in dried tilia tree leaves for each mass transition.

	0.01 mg/kg (n = 5)						0.4 mg/kg (n = 6)					
	Extraction yield (%)	RSD (%)	Matrix effects (%)	RSD (%)	Recovery (%)	RSD (%)	Extraction yield (%)	RSD (%)	Matrix effects (%)	RSD (%)	Recovery (%)	RSD (%)
**Anthracene_1**	83.1	5.7	675.6	13.7	478.4	11.4	70.2	8.7	738.6	12.6	473.2	8.5
**Anthracene_2**			789.8	11.2	568.9	9.6			689.3	11.8	442.3	10.8
**Anthracene_3**			[a]	[a]	[a]	[a]			627.4	11.2	378.1	9.3
**PCB-31**												
**Anthraquinone_1**	92.4	4.3	[b]	[b]	[b]	[b]	76.1	6.6	[b]	[b]	[b]	[b]
**Anthraquinone_2**			972.8	16.9	806.1	13.7			1894.5	40.4	512.0	9.4
**Anthraquinone_3**			848.4	16.7	739.4	12.7			1820.7	39.8	466.6	10.0
**1-Hydroxyanthraquinone_1**	111.1	5.7	1912.3	12.3	1875.6	11.7	78.8	2.7	1532.9	3.3	883.5	6.7
**1-Hydroxyanthraquinone_2**			1794.0	13.2	1664.2	11.7			1695.8	0.6	911.9	6.9
**1-Hydroxyanthraquinone_3**			667.6	31.1	656.8	13.4			1414.3	5.6	838.2	7.0
**2-Methylanthraquinone_1**	91.3	3.8	1057.7	14.6	864.0	13.4	80.1	3.8	1555.3	10.5	515.3	9.6
**2-Methylanthraquinone_2**			873.0	23.9	761.5	4.6			1678.0	12.0	570.2	8.0
**2-Methylanthraquinone_3**			1266.0	12.6	945.2	14.4			1733.2	10.1	575.0	9.5
**1,4-Dihydroxyanthraquinone_1**	160.4	13.2	[c]	[c]	[c]	[c]	84.9	3.8	1808.6	3.1	1297.2	6.2
**1,4-Dihydroxyanthraquinone_2**			[c]	[c]	[c]	[c]			1499.6	1.0	1114.6	5.9
**1,4-Dihydroxyanthraquinone_3**			[c]	[c]	[c]	[c]			1460.2	0.4	1003.6	6.3
**1,8-Dihydroxyanthraquinone_1**			[c]	[c]	[c]	[c]			1541.7	3.5	1146.2	5.8
**1,8-Dihydroxyanthraquinone_2**			[c]	[c]	[c]	[c]			1494.2	3.4	1111.9	6.6
**1,8-Dihydroxyanthraquinone_3**			[c]	[c]	[c]	[c]			1058.6	6.1	693.8	6.4
**1,2-Dihydroxyanthraquinone_1**	[d]	[d]	[e]	[e]	[e]	[e]	321.0	6.5	31.4	21.7	90.0	8.6
**1,2-Dihydroxyanthraquinone_2**			[e]	[e]	[e]	[e]			61.6	7.3	101.4	13.1
**1,2-Dihydroxyanthraquinone_3**			[e]	[e]	[e]	[e]			103.3	15.4	147.9	10.1
**Chrysophanol_1**	122.9	9.3	[c]	[c]	[c]	[c]	76.4	3.6	2018.4	20.4	1449.9	7.1
**Chrysophanol_2**			[c]	[c]	[c]	[c]			2128.0	19.5	1473.0	7.4
**Chrysophanol_3**			[c]	[c]	[c]	[c]			1596.1	17.8	989.9	6.2
**PCB-209**												

[a] Below limit of quantification. [b] The transition suffered from an unresolvable interference in extracts. [c] Absence of analyte protectants suppressed the signal. [d] Old age of column suppressed the signal. [e] Old age of column and the absence of analyte protectants suppressed the signal

Table 7. Linearity, limits of detection as well as quantification and replicability and reproducibility results for the validation in dried tilia trees for each mass transition.

	Linearity$_{Acetonitrile}$			Linearity$_{Matrix\text{-}matched}$					Replicability		Reproducibility	
	R^2	n	Range (mg/kg)	R^2	n	Range (mg/kg)	Limit of detection (µg/kg)	Limit of quantification (µg/kg)	$RSD_{0.01\ mg/kg}$ (%) n = 5	$RSD_{0.4\ mg/kg}$ (%) n = 6	$RSD_{0.01\ mg/kg}$ (%) n = 5	$RSD_{0.4\ mg/kg}$ (%) n = 5
**Anthracene_1**	0.9894	9	0 - 2	0.9978	6	0 - 1.6	1.8	3.0	6.6	7.6	20.4	16.4
**Anthracene_2**	0.9914	10	0 - 2	0.9989	6	0 - 1.6	1.5	2.5	5.9	9.9	21.8	18.9
**Anthracene_3**	0.9887	8	0 - 2	0.9864	7	0 - 1.6	11.0	18.3	[a]	9.0	137.6	20.8
**PCB-31**												
**Anthraquinone_1**	0.9952	9	0 - 2	[b]	[b]	0 - 1.6	[b]	[b]	[b]	[b]	[b]	[b]
**Anthraquinone_2**	0.9935	9	0 - 2	0.9967	6	0 - 1.6	0.2	0.4	7.0	6.1	19.5	12.7
**Anthraquinone_3**	0.9885	10	0 - 2	0.9951	6	0 - 1.6	2.5	4.1	7.4	7.3	27.4	16.8
**1-Hydroxyanthraquinone_1**	0.9564	11	0 - 2	0.9973	7	0 - 1.6	0.8	1.3	7.5	2.9	26.9	11.5
**1-Hydroxyanthraquinone_2**	0.9311	11	0 - 2	0.9966	7	0 - 1.6	0.6	1.0	5.5	2.3	30.7	14.4
**1-Hydroxyanthraquinone_3**	0.9745	11	0 - 2	0.9972	7	0 - 1.6	28.6	47.7	16.1	3.1	67.9	9.6
**2-Methylanthraquinone_1**	0.9844	11	0 - 2	0.9958	6	0 - 1.6	1.1	1.8	9.4	3.2	48.2	8.8
**2-Methylanthraquinone_2**	0.9895	11	0 - 2	0.9983	6	0 - 1.6	3.2	5.3	6.8	4.8	59.8	11.3
**2-Methylanthraquinone_3**	0.986	11	0 - 2	0.9943	6	0 - 1.6	7.4	12.3	7.8	4.0	57.0	10.0
**1,4-Dihydroxyanthraquinone_1**	0.825	11	0 - 2	0.9995	6	0 - 1.6	0.9	1.4	12.8	3.8	11.8	6.3
**1,4-Dihydroxyanthraquinone_2**	0.8457	11	0 - 2	0.9997	6	0 - 1.6	2.9	4.8	7.8	4.5	11.7	5.0
**1,4-Dihydroxyanthraquinone_3**	0.6361	11	0 - 2	0.9995	6	0 - 1.6	29.0	48.3	[a]	3.2	[a]	9.4
**1,8-Dihydroxyanthraquinone_1**	0.8556	11	0 - 2	0.9995	6	0 - 1.6	1.6	2.6	4.0	4.1	40.6	32.0
**1,8-Dihydroxyanthraquinone_2**	0.8389	11	0 - 2	0.9995	6	0 - 1.6	2.1	3.5	5.1	3.8	8.1	7.0
**1,8-Dihydroxyanthraquinone_3**	0.8151	11	0 - 2	0.9993	6	0 - 1.6	7.2	12.0	15.4	3.8	23.6	11.9
**1,2-Dihydroxyanthraquinone_1**	[c]	[c]	[c]	0.9919	6	0 - 1.6	10.8	17.9	25.8	6.5	223.6	78.2
**1,2-Dihydroxyanthraquinone_2**	[c]	[c]	[c]	0.9929	7	0 - 1.6	34.0	56.7	[a]	11.2	[a]	33.6
**1,2-Dihydroxyanthraquinone_3**	[c]	[c]	[c]	0.9863	7	0 - 1.6	55.7	92.8	[a]	11.0	[a]	41.9
**Chrysophanol_1**	[c]	[c]	[c]	0.9978	7	0 - 1.6	2.2	3.6	8.8	2.8	16.9	10.5
**Chrysophanol_2**	[c]	[c]	[c]	0.9933	7	0 - 1.6	1.9	3.2	9.7	2.5	22.6	9.2
**Chrysophanol_3**	[c]	[c]	[c]	0.996	7	0 - 1.6	14.3	23.9	26.7	10.5	57.5	9.5
**PCB-209**												

[a] Below limit of quantification. [b] The transition suffered from an unresolvable interference in the extracts. [c] Absence of analyte protectants suppressed the signal.

The dihydroxyanthraquinone chromatograms of the linearity measurements in pure acetonitrile were difficult to evaluate, due to the retention time concentration dependence, which shifted the corresponding peaks outside the measurement window depending on the injected amount. The last linearity matrix-matched standard at 2 mg/kg had to be eliminated as outlier. This was probably due to an operational error during extract preparation. The dynamic range upper limit of 1.6 mg/kg was high enough for these matrices, anyway. With respect to LOD and LOQ, all compounds had quantification limits well below AQ's MRL in dried leaves of 0.02 mg/kg. In replicability, all compounds had RSDs lower than 20% and, as excepted, the values were higher at 0.01 mg/kg. 12DHA was slightly higher at 26%. However, it is difficult to assess if such deviations originate during extraction or because of the long sequence at the instrument. Moreover, 12DHA's signals were observed during the extraction optimization experiments to depend strongly on the column age. Therefore, the high RSD values obtained are reasonable considering the operational variability. As for the extraction yield measurements, all values are overall acceptable. Interestingly, the comparison between percentages at 0.01 and 0.4 (mg/kg) yield statistically different results at 95% confidence for all compounds. This observation in theory contradicts the linearity determination. However, when the matrix effects are analysed, it can be observed that these have high deviation at both concentration levels and are statistically different at 95% confidence between the two levels. Since the same sample was extracted in replicability conditions, this probably means that liner and column conditions changed after the ~10 measurements between the two levels. This may be due to the high matrix-rich extracts with strong matrix effects. Such effect could have also influenced 12DHA. Nevertheless, since Equation 18 and Equation 20 correct for the extraction yield and the RSD is below 10%, 12DHA value at 0.4 mg/kg was accepted.

For the other matrices the extraction yield, the replicability and LOQ were determined in triplicates at a point of relevance for the values in the samples (Table 8 – 11), because fresh

commodities are analytically not as challenging as dried products due to the amount of co-extractives. To confirm that the linearity in fresh samples was maintained, the dynamic range was again checked with fresh leaves as model fresh matrix. Tilia tree leaves were again chosen as substitute model matrix.

Due to the reduced sample amounts of moss, validation could only be determined from different samples. That is why the RSD values for some analytes are relatively high. Overall, all values are acceptable. No signal of 12DHA could be observed whatsoever, maybe due to column aging. For the woody parts of the plant no validation was carried out because the matrix was minimal. The values from another matrix were applied to branches and walnut shells. The material of choice was moss, because the dilution is similar (only a difference of factor 2) and the extracts are clean (that is, not too matrix-concentrated).

The extraction yields of the analytes in walnut kernels were significantly lower than in other matrices, although still acceptable. This is probably due to their partial retention in the lipidic fifth phase originating from the high fatty acid content of walnuts. 12DHA showed again a yield well above 140%. This alone does not pose a problem for quantification, because all measurements are corrected for the extraction yield. However, the RSD is also higher than 20%. Therefore, 12DHA was not quantified in walnut kernels.

The linearity of 1HA in dried and fresh leaves was also checked in a higher magnitude with 7 $Spike_{Extraction}$ standards at the levels 0, 2, 6, 10, 14, 18 and 22 (mg/kg), due to the more concentrated values of this compound in the walnut leaf samples. It reached up to 14 mg/kg.

Table 8. Linearity, limits of detection and quantification, replicability as well as extraction yield validation results for fresh tilia tree leaves for each mass transition.

	R^2	n	Range (mg/kg)	Limit of detection (µg/kg)	Limit of quantification (µg/kg)	$RSD_{0.1\ mg/kg}$ (%) n = 3	Extraction yield (%) n = 3	RSD (%) n = 3
**Anthracene_1**	0.9928	8	0 - 1.056	0.7	1.1	0.3	77.5	1.7
**Anthracene_2**	0.9917	8	0 - 1.056	1.0	1.6	1.5		
**Anthracene_3**	0.9935	8	0 - 1.056	6.0	10.0	3.8		
**PCB-31**								
**Anthraquinone_1**	[a]	[a]	0 - 1.056	[a]	[a]	[a]	86.4	3.6
**Anthraquinone_2**	0.997	7	0 - 1.056	0.2	0.3	2.2		
**Anthraquinone_3**	0.994	8	0 - 1.056	2.1	3.4	5.3		
**1-Hydroxyanthraquinone_1**	0.9983	7	0 - 1.056	0.4	0.6	3.0	84.3	3.2
**1-Hydroxyanthraquinone_2**	0.999	7	0 - 1.056	0.3	0.6	3.5		
**1-Hydroxyanthraquinone_3**	0.9981	7	0 - 1.056	0.9	1.5	3.2		
**2-Methylanthraquinone_1**	0.9986	7	0 - 1.056	0.4	0.7	1.7	85.4	1.5
**2-Methylanthraquinone_2**	0.9993	7	0 - 1.056	1.4	2.3	1.6		
**2-Methylanthraquinone_3**	0.9985	7	0 - 1.056	0.6	1.1	1.6		
**1,4-Dihydroxyanthraquinone_1**	0.9965	7	0 - 1.056	0.4	0.7	2.2	78.9	2.2
**1,4-Dihydroxyanthraquinone_2**	0.9983	7	0 - 1.056	0.9	1.4	2.1		
**1,4-Dihydroxyanthraquinone_3**	0.991	8	0 - 1.056	9.5	15.8	3.7		
**1,8-Dihydroxyanthraquinone_1**	0.997	7	0 - 1.056	0.2	0.3	2.3		
**1,8-Dihydroxyanthraquinone_2**	0.9972	7	0 - 1.056	0.8	1.4	1.8		
**1,8-Dihydroxyanthraquinone_3**	0.9899	8	0 - 1.056	6.0	10.0	1.7		
**1,2-Dihydroxyanthraquinone_1**	0.9865	6	0 - 0.864	1.9	3.1	5.1	142.5	3.5
**1,2-Dihydroxyanthraquinone_2**	0.9621	7	0 - 1.056	12.6	21.0	2.8		
**1,2-Dihydroxyanthraquinone_3**	0.9954	7	0 - 1.056	26.1	43.5	9.1		
**Chrysophanol_1**	0.9958	8	0 - 1.056	1.1	1.8	1.7	75.7	1.2
**Chrysophanol_2**	0.9948	8	0 - 1.056	0.7	1.2	3.7		
**Chrysophanol_3**	0.9961	8	0 - 1.056	9.2	15.4	5.2		
**PCB-209**								

[a] The transition suffered from an unresolvable interference in the extracts.

Table 9. Linearity, limits of detection and quantification, replicability as well as extraction yield validation results for moss for each mass transition determined at 0.1 mg/kg and for 1-hydroxyanthraquinone at 4 mg/kg.

0.1 mg/kg (1-hydroxyanthraquinone 4 mg/kg)	Limit of detection (µg/kg)	Limit of quantification (µg/kg)	RSD (%) n = 2	Extraction yield (%) n = 2	RSD (%) n = 2
Anthracene_1	1.6	2.6	2.7	83.2	5.9
Anthracene_2	1.2	2.0	3.8		
Anthracene_3	7.4	12.3	10.1		
PCB-31					
Anthraquinone_1	[a]	[a]	6.2	107.3	9.0
Anthraquinone_2	0.6	1.1	0.0		
Anthraquinone_3	0.8	1.4	1.7		
1-Hydroxyanthraquinone_1	0.3	0.5	0.4	65.7	24.1
1-Hydroxyanthraquinone_2	0.1	0.2	1.8		
1-Hydroxyanthraquinone_3	1.1	1.8	0.3		
2-Methylanthraquinone_1	0.9	1.5	18.4	107.2	5.9
2-Methylanthraquinone_2	2.1	3.4	10.4		
2-Methylanthraquinone_3	1.9	3.2	13.3		
1,4-Dihydroxyanthraquinone_1	1.3	2.1	13.4	91.4	6.1
1,4-Dihydroxyanthraquinone_2	8.1	13.6	5.7		
1,4-Dihydroxyanthraquinone_3	56.3	93.8	3.7		
1,8-Dihydroxyanthraquinone_1	0.9	1.4	10.6		
1,8-Dihydroxyanthraquinone_2	0.9	1.4	12.7		
1,8-Dihydroxyanthraquinone_3	6.0	10.0	1.0		
1,2-Dihydroxyanthraquinone_1	[b]	[b]	[b]	[b]	[b]
1,2-Dihydroxyanthraquinone_2	[b]	[b]	[b]		
1,2-Dihydroxyanthraquinone_3	[b]	[b]	[b]		
Chrysophanol_1	4.0	6.7	36.1	93.9	15.0
Chrysophanol_2	2.4	4.0	33.7		
Chrysophanol_3	18.8	31.3	28.0		
PCB-209					

[a] The transition suffered from an unresolvable interference in the extracts. [b] No signal observable due to column aging.

Table 10. Linearity, limits of detection and quantification, replicability as well as extraction yield validation results for walnut husks for each mass transition determined at 0.5 mg/kg.

0.5 mg/kg	Limit of detection (µg/kg)	Limit of quantification (µg/kg)	RSD (%) n = 3	Extraction yield (%) n = 3	RSD (%) n = 3
**Anthracene_1**	0.2	0.3	3.4	84.2	4.3
**Anthracene_2**	0.2	0.3	4.0		
**Anthracene_3**	1.4	2.4	5.5		
**PCB-31**					
**Anthraquinone_1**	[a]	[a]	3.2	99.5	2.7
**Anthraquinone_2**	0.1	0.1	3.4		
**Anthraquinone_3**	0.1	0.1	1.7		
**1-Hydroxyanthraquinone_1**	0.4	0.6	4.7	96.9	4.2
**1-Hydroxyanthraquinone_2**	0.3	0.5	4.4		
**1-Hydroxyanthraquinone_3**	0.4	0.7	4.3		
**2-Methylanthraquinone_1**	0.1	0.1	5.0	96.7	4.8
**2-Methylanthraquinone_2**	0.2	0.3	4.5		
**2-Methylanthraquinone_3**	0.2	0.3	5.0		
**1,4-Dihydroxyanthraquinone_1**	0.7	1.2	5.4	100.8	5.3
**1,4-Dihydroxyanthraquinone_2**	0.2	0.4	5.1		
**1,4-Dihydroxyanthraquinone_3**	5.1	8.5	4.1		
**1,8-Dihydroxyanthraquinone_1**	0.5	0.8	5.7		
**1,8-Dihydroxyanthraquinone_2**	0.8	1.4	6.4		
**1,8-Dihydroxyanthraquinone_3**	3.5	5.8	6.8		
**1,2-Dihydroxyanthraquinone_1**	2.7	4.5	0.7	95.1	6.7
**1,2-Dihydroxyanthraquinone_2**	7.9	13.2	2.6		
**1,2-Dihydroxyanthraquinone_3**	9.0	15.0	3.9		
**Chrysophanol_1**	0.4	0.6	10.3	105.6	8.8
**Chrysophanol_2**	0.3	0.4	9.0		
**Chrysophanol_3**	1.3	2.2	7.8		
**PCB-209**					

[a] The transition suffered from an unresolvable interference in the extracts.

Table 11. Linearity, limits of detection and quantification, replicability as well as extraction yield validation results for walnut kernels for each mass transition determined at 0.1 mg/kg.

0.1 mg/kg	Limit of detection (µg/kg)	Limit of quantification (µg/kg)	RSD (%) n = 3	Extraction yield (%) n = 3	RSD (%) n = 3
**Anthracene_1**	0.4	0.6	5.6	66.4	6.6
**Anthracene_2**	0.5	0.8	4.8		
**Anthracene_3**	4.3	7.1	10.2		
**PCB-31**					
**Anthraquinone_1**	[a]	[a]	9.6	66.6	8.9
**Anthraquinone_2**	0.9	1.5	6.7		
**Anthraquinone_3**	0.6	1.1	10.9		
**1-Hydroxyanthraquinone_1**	0.3	0.4	7.6	78.4	8.1
**1-Hydroxyanthraquinone_2**	0.3	0.5	8.5		
**1-Hydroxyanthraquinone_3**	0.2	0.3	7.9		
**2-Methylanthraquinone_1**	0.2	0.3	9.6	67.8	9.5
**2-Methylanthraquinone_2**	0.8	1.4	9.7		
**2-Methylanthraquinone_3**	1.5	2.4	9.5		
**1,4-Dihydroxyanthraquinone_1**	0.4	0.7	11.0	86.0	9.2
**1,4-Dihydroxyanthraquinone_2**	4.7	7.9	11.4		
**1,4-Dihydroxyanthraquinone_3**	10.6	17.7	10.4		
**1,8-Dihydroxyanthraquinone_1**	0.5	0.8	10.8		
**1,8-Dihydroxyanthraquinone_2**	2.2	3.7	10.6		
**1,8-Dihydroxyanthraquinone_3**	4.7	7.9	4.0		
**1,2-Dihydroxyanthraquinone_1**	6.7	11.2	19.7	330.4	26.5
**1,2-Dihydroxyanthraquinone_2**	8.3	13.9	47.3		
**1,2-Dihydroxyanthraquinone_3**	24.0	40.0	15.5		
**Chrysophanol_1**	3.2	5.3	9.6	67.7	8.5
**Chrysophanol_2**	0.7	1.2	5.5		
**Chrysophanol_3**	14.5	24.1	11.0		
**PCB-209**					

[a] The transition suffered from an unresolvable interference in the extracts.

SI6 – Principal component analysis

To discern the variables that best fit the variance, the *J. regia* leaf data were modelled via principal component analysis (PCA). The variables chosen were the concentrations of the analytes, maximal and minimum temperature, precipitation, wind speed, pressure and date. Circumference at breast height (CBH), latitude, longitude, altitude and distance to a city were not included due to their constancy throughout the replicate sampling in time at each location. This characteristic can hinder the PCA, since the model looks for the direction with highest variance. In that regard, the samples of Sarajevo and Seona that were taken using replicate sampling in space were aggregated around the mean for each tree. For pre-processing, features were auto scaled to 0 mean and unit variance. PCA was first run with four components to visually detect outlying samples and eliminate them from the model. The number of principal components selected to run the PCA was based on the cumulative explained variance levelling off. To draw the biplots, the loadings were scaled up using the range of the corresponding scores, which were coloured depending on the location of the weather station (Figure 12).

The difference between Sarajevo and the Bosnian mountains is not clear because the samples from the capital appear close to the average and lie, therefore, in the centre of the data cloud. On the other hand, PC1 clearly separates the samples according to the countries. Germany correlates with AQ and 1HA but also with precipitation, whereas samples from Spain and Bosnia and Herzegovina are related to the derivatives and temperatures. This distinction disappears for the other principal components and no clear behaviour can be observed. PC4 seems to separate Adlhausen samples from all the other based on anticorrelation with AQ, 1HA, 148DHA, chrysophanol, precipitation and temperature. However, the percentage of variance explained is less than 10% and the differentiation is not clear. The classification of PC1 clearly indicates that precipitation and temperature are the determining factors of the two peaks observed in the distribution of AQ and 1HA. This may indicate that lower temperatures help to reduce the airborne more volatile AQ and 1HA content, aided by wet scavenging, which deposits on the leaf surface and adds to the amount

that the plant already endogenously produces. However, such explanation does not account for the significant difference in concentration scale between AQ and 1HA since such high amounts of 1HA are unlikely to originate alone from the atmosphere. In that regard, one has to bear in mind that PC1 only explains ~30% of the variance, which means that other underlaying factors also have an influence. Therefore, none of these variables alone can fit the data, and maybe parameters not included in the PCA also have a determining influence.

The loading analysis reveals that PC1PC3, PC1PC4 and PC3PC4 all show a strong correlation between AQ and 1HA, whereas in the other biplots their angle opens to *ca*. 60º. This behaviour clearly matches the conclusions from the jointplots. When AQ and 1HA correlate, their angle with the other derivatives lies between 90º and 180º, although chrysophanol tends to appear closer to orthogonality. Such dependence remains for AQ in the other biplots, while 1HA moves closer to the derivatives at angles smaller than 90º. The correlation with the atmosphere is somewhat more ambiguous. When AQ and 1HA are correlated, PC1PC3 shows no dependence with neither precipitation nor temperature, while PC1PC4 suggests a possible correlation with rain and PC3PC4 with temperature. With no AQ-1HA correlation, AQ exhibits again an ambiguous behaviour. However, instead of tilting in some biplots towards correlation, it moves into anti correlation angles with weather. On the other hand, 1HA keeps gravitating between orthogonality and correlation. However, the angles now seem much closer. As for the derivatives with respect to the weather conditions, they have a very vague relationship. This ambiguity is much clearer when analysing the samples from replicate sampling in space. Leaves that have had the exact same weather conditions show remarkedly high heterogeneity in all analyte dimensions. As for the feature ‘time’, no clear relationship can be determined. However, angles seem to be in general closer to 90º, although chrysophanol shows a certain correlation. Lastly, the variables pressure and wind speed do not seem to contain much information, since they tend to correlate or anticorrelate with other weather parameters depending on anti- or cyclonic conditions.

Overall, the PCA has not unambiguously clarified the underlying relationships. The main conclusion that can be drawn from it is that precipitation and low temperature does seem to have an impact on AQ concentration.

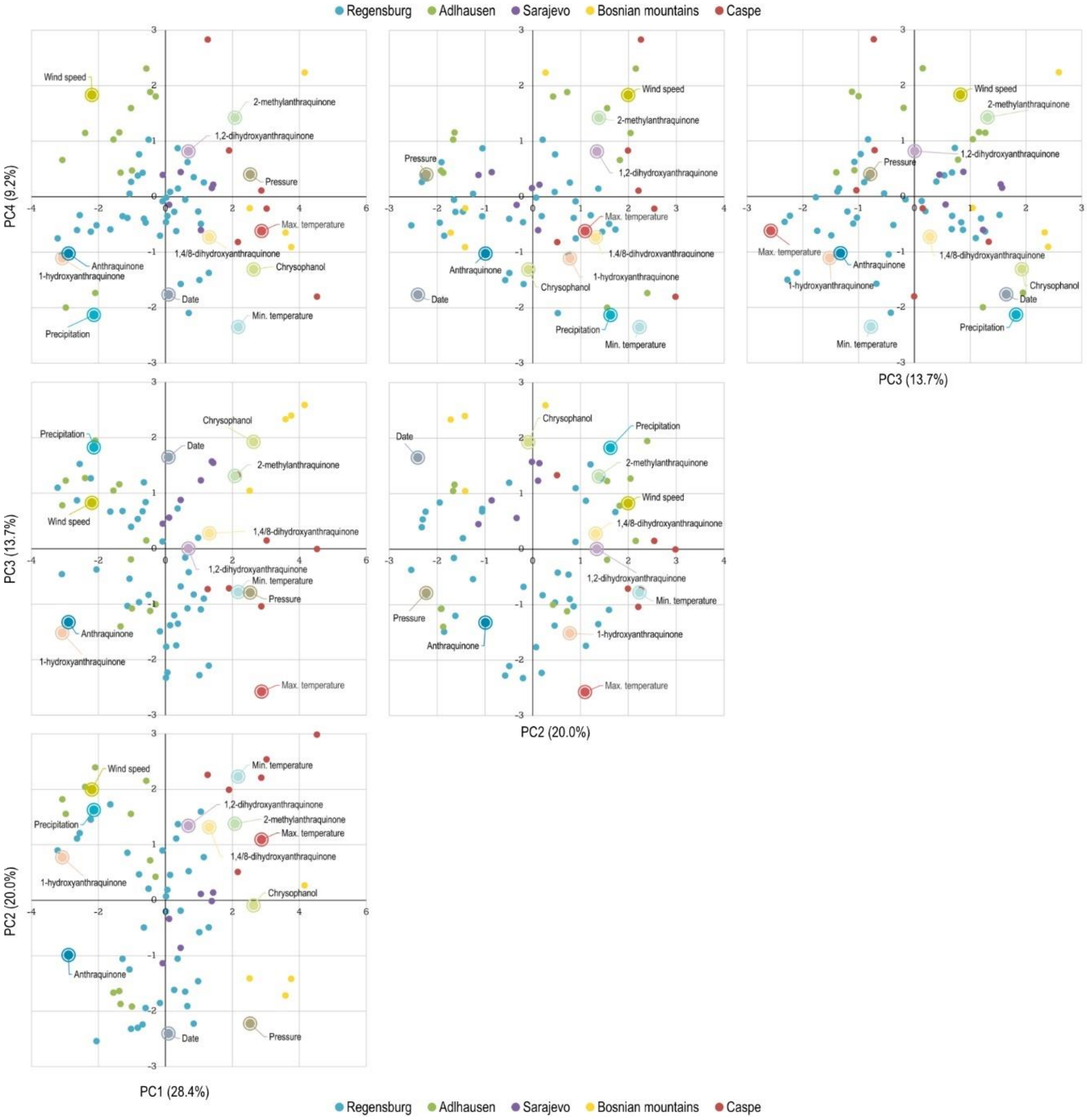

Figure 12. Biplots from the principal component analysis of the *J. regia* leaves.

SI7 – Fresh samples

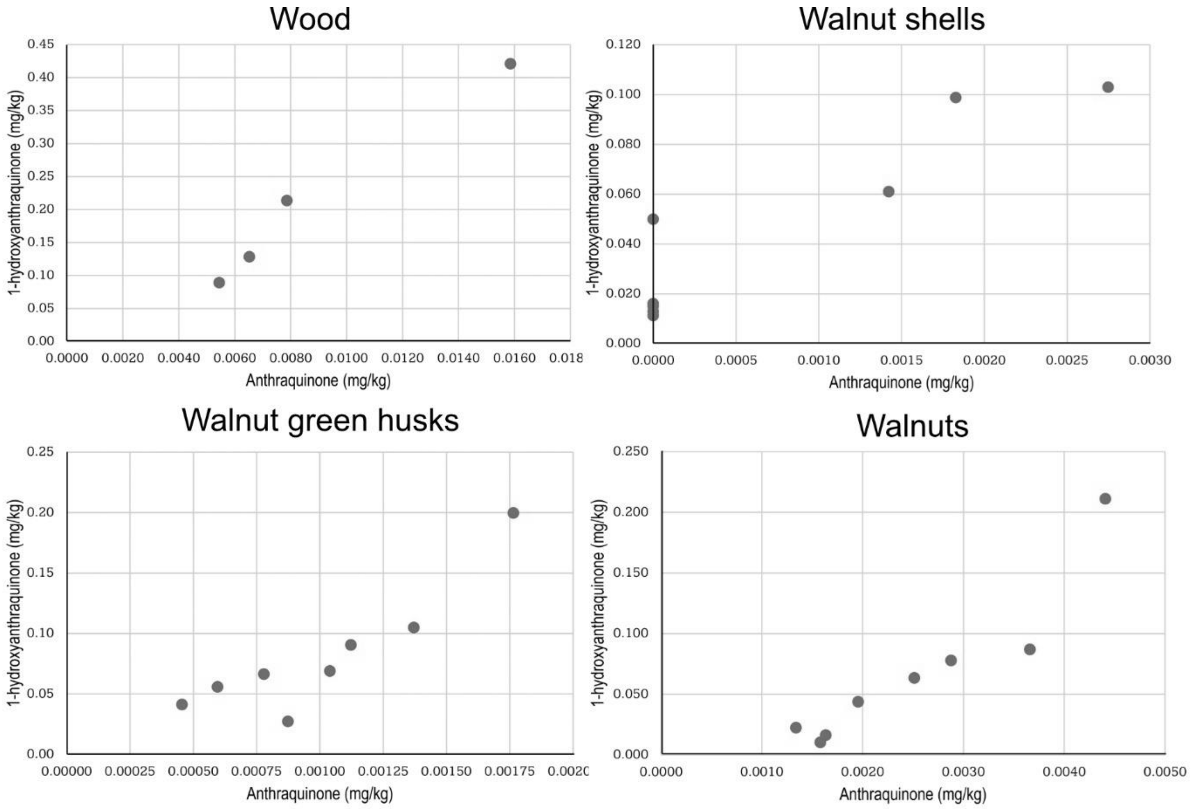

Figure 13. Correlation plot between the concentration in mg/kg of 1-hydroxyanthraquinone *vs*. anthraquinone in wood (top right), walnut shells (top left), walnut green husks (bottom left) and walnuts (bottom right).

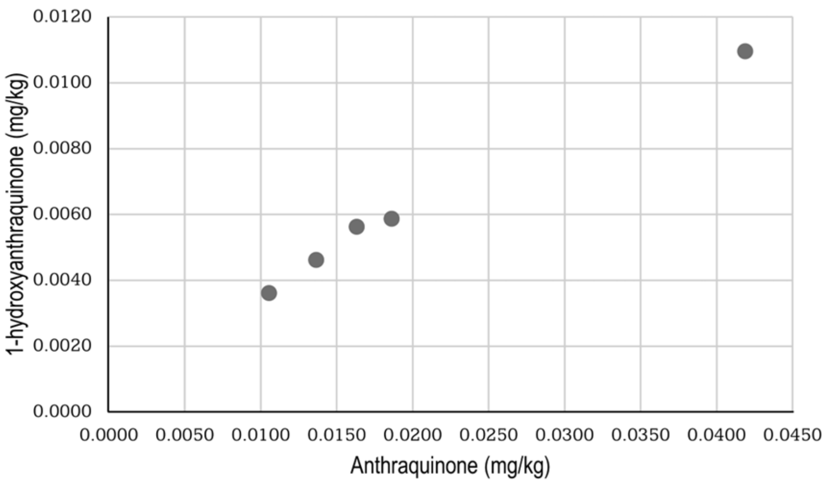

Figure 14. Correlation plot for concentration in mg/kg of 1-hydroxyanthraquinone *vs*. anthraquinone in moss.

Replicate sampling in space

From a visual analysis (Figure 15), no correlation between positions located higher as well as on the surface of the treetop and concentration of the analytes can be determined. This is however not a definitive answer, because treetops were relatively small and not dense enough to show significant gradients in radiation. Nevertheless, other remarks can be made from this figure. For example, leaves from Seona 'BergLoc1' are the only ones of all analysed samples that show AQ content lower than 0.01 mg/kg. In fact, the outlying behaviour of this tree does not end there. 1HA content is also the lowest quantified, whereas chrysophanol shows the highest. The other derivatives seem constant with respect to other samples. The hypothesis of a reduced atmospheric ad- and absorption, mainly of AQ and 1HA, in the isolated village in the Bosnian mountains is again unlikely, since the most isolated tree of this work, found around 10 km away from Seona at the location 'BergLoc2' shows (although still low compare with other samples) higher contents of AQ and 1HA, while the other derivatives remain constant with respect to Seona. This indicates that there is something defining about the location 'BergLoc1'. It must have to do with the area and not specifically the plant, because the other 2 trees standing close to the central one, 'BergLoc1area1' and 'BergLoc1area2', also show remarkedly low contents of both AQ and 1HA. This suggests that there might indeed exist an additional location specific parameter that could unveil why the German samples are different. From these characteristic low values, one could hypothesize that the shikimic acid is, for some reason, hindered in the Bosnian mountains, whereas the polyketide pathway is more active, as can be seen with the higher chrysophanol content. However, 2MA would be expected to be also more diluted since it is an important product of SA way. More information is needed to accurately discern the underlying processes. Another remark that can be made from Figure 15 is that the linearity between AQ and 1HA is present in all replicates in space for all trees. Moreover, the derivatives seem to also correlate with each other both in variance and

concentration. Roughly, the same 2 pairs as before can be done. On the one hand, 2MA and 12DHA, which are the theoretical SA derivatives. On the other, 148DHA and chrysophanol from the polyketide pathways, which seem to appear more concentrated than the SA derivatives.

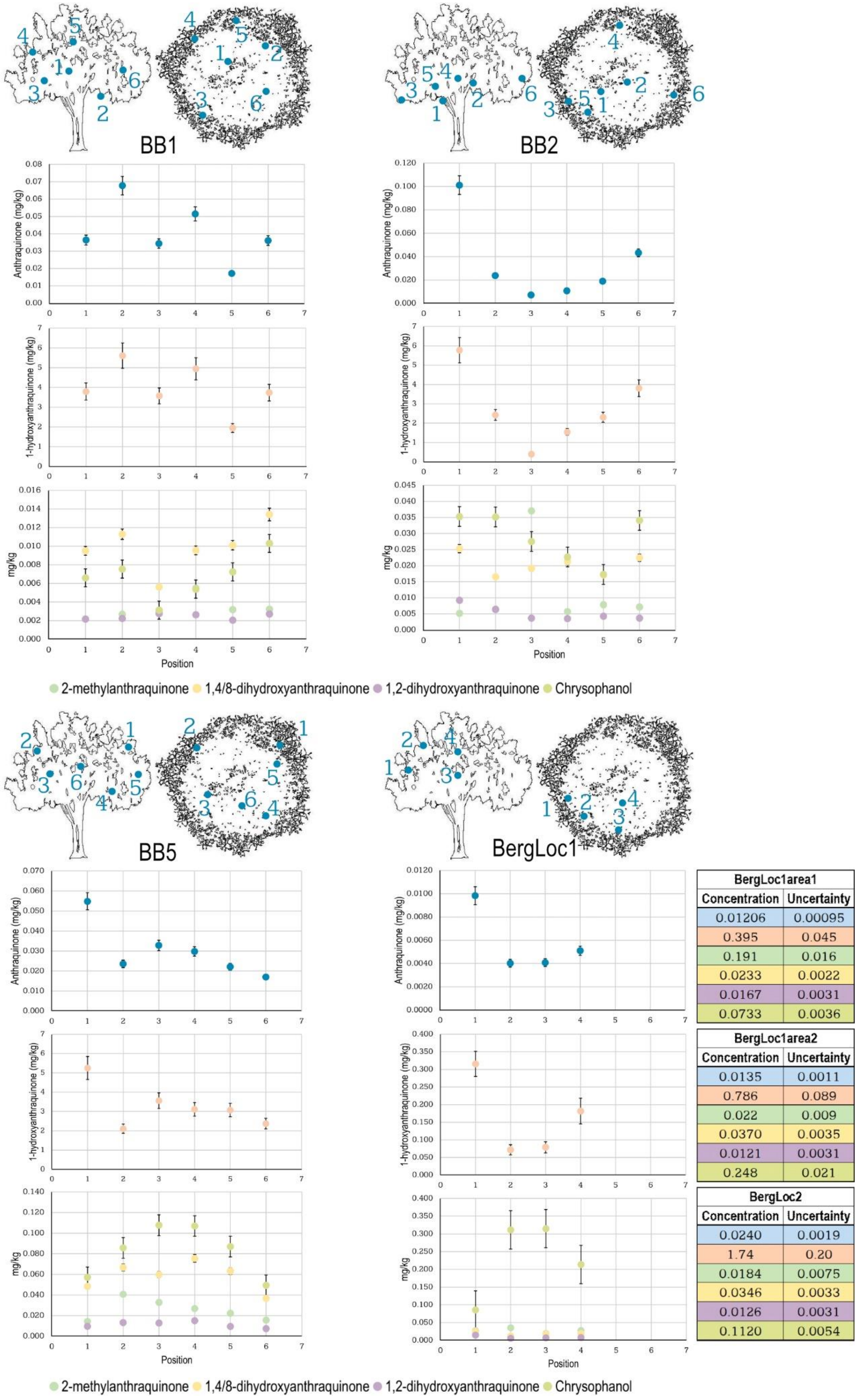

BergLoc1area1	
Concentration	Uncertainty
0.01206	0.00095
0.395	0.045
0.191	0.016
0.0233	0.0022
0.0167	0.0031
0.0733	0.0036

BergLoc1area2	
Concentration	Uncertainty
0.0135	0.0011
0.786	0.089
0.022	0.009
0.0370	0.0035
0.0121	0.0031
0.248	0.021

BergLoc2	
Concentration	Uncertainty
0.0240	0.0019
1.74	0.20
0.0184	0.0075
0.0346	0.0033
0.0126	0.0031
0.1120	0.0054

Figure 15. Concentration (in mg/kg) of the analytes in the samples from the replicates in space from Bosnia. 2D tree drawing obtained from Pintereset/designscad.com.
